# NR5A2 connects zygotic genome activation to the first lineage segregation in totipotent embryos

**DOI:** 10.1038/s41422-023-00887-z

**Published:** 2023-11-07

**Authors:** Fangnong Lai, Lijia Li, Xiaoyu Hu, Bofeng Liu, Ziqi Zhu, Ling Liu, Qiang Fan, Huabin Tian, Kai Xu, Xukun Lu, Qing Li, Kong Feng, Lijuan Wang, Zili Lin, Hongyu Deng, Jinsong Li, Wei Xie

**Affiliations:** 1 Center for Stem Cell Biology and Regenerative Medicine, MOE Key Laboratory of Bioinformatics, New Cornerstone Science Laboratory, School of Life Sciences, Tsinghua University, Beijing, China; 2 Tsinghua-Peking Center for Life Sciences, Beijing, China; 3 CAS Key Laboratory of Infection and Immunity, Institute of Biophysics, Chinese Academy of Sciences, Beijing, China; 4 State Key Laboratory of Cell Biology, Shanghai Key Laboratory of Molecular Andrology, Shanghai Institute of Biochemistry and Cell Biology, Center for Excellence in Molecular Cell Science, Chinese Academy of Sciences, University of Chinese Academy of Sciences, Shanghai, China; 5 College of Animal Science and Technology College, Beijing University of Agriculture, Beijing, China

**Keywords:** Pluripotency, Transcriptional regulatory elements

## Abstract

Zygotic genome activation (ZGA) marks the beginning of the embryonic program for a totipotent embryo, which gives rise to the inner cell mass (ICM) where pluripotent epiblast arises, and extraembryonic trophectoderm. However, how ZGA is connected to the first lineage segregation in mammalian embryos remains elusive. Here, we investigated the role of nuclear receptor (NR) transcription factors (TFs), whose motifs are highly enriched and accessible from the 2-cell (2C) to 8-cell (8C) stages in mouse embryos. We found that NR5A2, an NR TF strongly induced upon ZGA, was required for this connection. Upon *Nr5a2* knockdown or knockout, embryos developed beyond 2C normally with the zygotic genome largely activated. However, 4–8C-specific gene activation was substantially impaired and *Nr5a2*-deficient embryos subsequently arrested at the morula stage. Genome-wide chromatin binding analysis showed that NR5A2-bound *cis*-regulatory elements in both 2C and 8C embryos are strongly enriched for B1 elements where its binding motif is embedded. NR5A2 was not required for the global opening of its binding sites in 2C embryos but was essential to the opening of its 8C-specific binding sites. These 8C-specific, but not 2C-specific, binding sites are enriched near genes involved in blastocyst and stem cell regulation, and are often bound by master pluripotency TFs in blastocysts and embryonic stem cells (ESCs). Importantly, NR5A2 regulated key pluripotency genes *Nanog* and *Pou5f1/Oct4*, and primitive endoderm regulatory genes including *Gata6* among many early ICM genes, as well as key trophectoderm regulatory genes including *Tead4* and *Gata3* at the 8C stage. By contrast, master pluripotency TFs NANOG, SOX2, and OCT4 targeted both early and late ICM genes in mouse ESCs. Taken together, these data identify NR5A2 as a key regulator in totipotent embryos that bridges ZGA to the first lineage segregation during mouse early development.

## Introduction

Following fertilization, the global transcription of the embryo is initially silenced.^1^ After a few cell cycles, thousands of zygotic transcripts are produced, known as zygotic genome activation (ZGA).^2^ ZGA can be divided into two waves: the minor ZGA and major ZGA, which occur at the middle 1-cell (1C) stage and late 2-cell (2C) stage in mice, respectively.^3^ By the 8-cell (8C) stage, blastomeres could still contribute to all lineages in chimeric mice^4–6^ and were considered as totipotent states in a broad definition.^7^ At the late 8C stage, the mouse embryo starts to compact and further divide, ultimately giving rise to two lineages: inner cell mass (ICM) which includes the founder of pluripotent cells, and trophectoderm (TE), which develops to the extraembryonic layer.^8^ ICM subsequently gives rise to *Nanog*-expressing pluripotent epiblast and *Gata6*-expressing primitive endoderm (PrE) at E4.5.^9^ The epiblast cells are the origin of all future embryonic lineages.^10^ The naïve pluripotency can be captured in vitro through the derivation of embryonic stem cells (ESCs) from ICM. The core pluripotency transcription factors (TFs) NANOG, SOX2 and OCT4 (NSO) cooperatively regulate the intrinsic pluripotency transcription network.^9,11–13^ Besides, ESCs also express “ancillary” pluripotency regulators, such as ESRRB, KLF4, SALL4, and TBX3, which can enhance the pluripotency network.^8^ Despite remarkable progress in understanding the pluripotency regulation, how ZGA is connected to the pluripotency establishment remains elusive to date.

Recently, with the advance of ultra-sensitive chromatin analysis approaches, dramatic reprogramming of chromatin landscapes has been revealed in early mammalian embryos.^2,14^ These chromatin maps also identified putative regulatory elements that exhibit highly dynamic activities in early embryogenesis, which may recruit master TFs to regulate the transcription network. However, the identities of these TFs and how their interactions with *cis*-regulatory elements govern transcription circuitry remain poorly understood. Notably, TFs can be inferred from motifs embedded in regulatory elements identified by assay for transposase-accessible chromatin with high-throughput sequencing (ATAC-seq) or DNase I hypersensitive site sequencing.^15,16^ Interestingly, the motifs of nuclear receptor (NR) family factors such as NR5A2 and RARG are overrepresented in the accessible putative regulatory elements in mouse 2–8C embryos.^17^ NRs are structurally conserved, ligand-dependent TFs, with diverse functions in cell proliferation, metabolism, stem cell pluripotency, and homeostasis.^18^ For example, RARG was shown to participate in the 2C-like-cell program,^19^ although its deficiency alone was compatible with mouse early development likely due to redundancy from the RAR family.^20,21^ NR5A2 was suggested to bind phosphatidyl inositol, although murine NR5A2 appears to have lost the ligand binding ability and becomes ligand-independent during evolution.^22^ NR5A2 is also closely linked to pluripotency regulation. *Nr5a2* is essential for early embryogenesis and regulates the expression of *Pou5f1* in the epiblast.^22–24^ In mouse ESCs (mESCs), NR5A2 is regulated by canonical Wnt/β-Catenin signaling and in turn directly controls pluripotency genes *Oct4*, *Nanog*, and *Tbx3*.^25^ Furthermore, NR5A2 can induce epiblast stem cell into ground-state pluripotency,^26^ and replace OCT4 in iPSC reprogramming.^24^ ESRRB and NR5A2 cooperatively play essential roles as auxiliary factors of OCT4 and SOX2 for the core-pluripotent network in mESCs.^27^ These studies underscore the potential roles of NR TFs in early development and pluripotency regulation. However, how these TFs interact with chromatin and govern transcription circuitry in vivo remains poorly understood.

Here, we screened for NR TFs that may potentially function in early embryos after ZGA and identified NR5A2 as a critical regulator for mouse early development. Knockdown (KD) or knockout (KO) of *Nr5a2* impaired the activation of 4–8C genes and led to embryonic arrest around the morula stage. Using CUT&RUN, we profiled NR5A2 chromatin occupancy at the 2C and 8C stages, and revealed that NR5A2 directly activated 4–8C gene programs including a set of key pluripotency genes. Furthermore, it opened a set of regions specifically activated at the 8C stage and bound by master pluripotency TFs such as NSO in blastocyst or mESC. These data suggest that NR5A2 plays a critical role in regulating early embryo development by connecting ZGA to the first lineage specification program.

## Results

### NR TFs regulate mouse early development

**Fig. 1:**
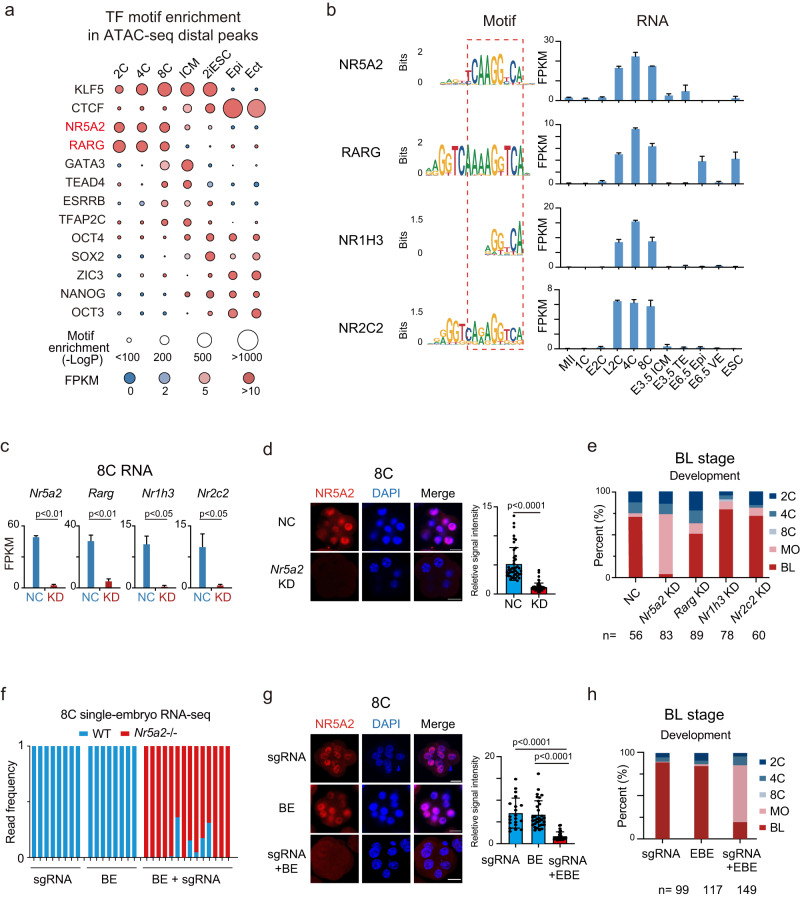
Both KD and KO of *Nr5a2* led to morula arrest. **a** TF motifs identified from distal ATAC-seq peaks^17,28^ in mouse early embryos. Sizes of circles indicate the –Log*P* value. Expression levels of TFs are color-coded. Epi, E6.5 Epiblast; Ect, E7.5 ectoderm. **b** Sequence logos of the binding motifs of NR5A2, RARG, NR1H3, and NR2C2 (left), and RNA expression (FPKM) of *Nr5a2*, *Rarg*, *Nr1h3*, and *Nr2c2* (right). VE, visceral endoderm. The error bars denote the standard deviations of two biological replicates of RNA-seq. **c** Bar charts showing the expression of *Nr5a2*, *Rarg*, *Nr1h3*, and *Nr2c2* in the negative control (NC) group (injected with negative control siRNA) and siRNA targeting group based on RNA-seq. The error bars denote the standard deviations of two biological replicates of RNA-seq. **d** Immunofluorescence of NR5A2 (red) and DAPI (blue) in mouse 8C embryos after KD of *Nr5a2* (left). Scale bars: 20 μm. Quantification of NR5A2 signal intensity (relative to DAPI) and *P v*alues (*t*-test, two-sided) are shown (right, *n* = 12–13 embryos). Each dot represents a single blastomere. **e** Bar plots showing the developmental rates of NC group, *Nr5a2* KD group, *Rarg* KD group, *Nr1h3* KD group, and *Nr2c2* KD group at the blastocyst stage (E4.5). MO, morula; BL, blastocyst. **f** Bar charts showing the percentage of WT *Nr5a2* (blue) and base-edited *Nr5a2* (red) reads from RNA-seq at the 8C stage after injection of *Nr5a2* sgRNA only, base-editor (BE) mRNA only, and both sgRNA and BE mRNA. Each bar represents a single embryo. **g** Immunofluorescence of NR5A2 (red) and DAPI (blue) in mouse 8C embryos after injection of sgRNA only, BE mRNA only, and both sgRNA and BE mRNA (left). Scales bars: 20 μm. Quantification of NR5A2 signal intensity (relative to DAPI) and *P v*alues (*t*-test, two-sided) are shown (right, *n* = 6–8 embryos). Each dot represents a single blastomere. **h** Bar plots showing the developmental rates at the blastocyst stage (E4.5) after injection of sgRNA only, BE mRNA only, and both sgRNA and BE mRNA.

To identify putative key TFs functioning between ZGA and the first lineage segregation in mouse early development, we firstly investigated the regulatory elements from our previous ATAC-seq data,^17,28^ which showed strong enrichment of motifs of NR TFs, including NR5A2 and RARG, from the 2–8C stages (Fig. 1a). Consistently, these two TFs were highly activated upon ZGA but their expression declined after the 8C stage (Fig. 1b, right). As a comparison, pluripotency TFs NSO’s motifs were enriched in ICMs and mESCs, paralleled by their expression dynamics (Fig. 1a). NR5A2 protein was undetectable in oocytes under our condition, consistent with its weak transcription levels, but was strongly induced at the 2C stage using immunofluorescence (Supplementary information, Fig. S1a). Given that NR family members often share similar motif sequences (Fig. 1b, left),^29^ we asked whether the NR family TFs may play redundant roles during early development. RNA-sequencing (RNA-seq) analysis showed that two additional NR family TFs *Nr1h3* and *Nr2c2* were also highly expressed from 2–8C, and exhibited similar binding motifs (Fig. 1b). Therefore, we first performed quadruple knockdown (4KD) against these four NR factors by injecting combined siRNAs into zygotes (Supplementary information, Fig. S1b, left). Remarkably, the 4KD embryos developed to 8C at a lower rate and barely developed to blastocysts, with most embryos arrested at the morula stage (Supplementary information, Fig. S1b, middle and right). RNA-seq confirmed the KD efficiency of these NR TFs at the 4C and 8C stages (Supplementary information, Fig. S1c). In particular, 4C-specifically activated genes and 8C-specifically activated genes (Materials and Methods; Supplementary information, Tables S1 and S2) were substantially downregulated upon KD of the 4 NR TFs (Supplementary information, Fig. S1d). Taken together, these data indicate that NR family TFs play important roles in early development and transcription regulation at the 4–8C stages.

### KD or KO of *Nr5a2* leads to developmental arrest at the morula stage

To ask which NR TF plays a more critical role in early development, we removed each one of the 4 siRNAs in the combined siRNAs and evaluated the developmental rates at E4.5 (blastocyst) (Supplementary information, Fig. S2a, b). Remarkably, when knocking down these 4 NR TFs, leaving out the siRNA targeting *Nr5a2*, but not *Nr1h3* or *Nr2c2*, substantially “rescued” the morula arrest phenotype of the 4KD embryos (Supplementary information, Fig. S2c). Leaving out *Rarg* siRNA had a moderate “rescue” effect (Supplementary information, Fig. S2c). Consistently, leaving out *Nr5a2* siRNA also restored normal 4C and 8C transcriptional programs (Supplementary information, Fig. S2d), indicating that NR5A2 is the main TF responsible for the 4KD phenotype. To further confirm this result, we performed a single KD for each of the 4 NR TFs (Fig. 1c). Indeed, knocking down *Nr5a2* alone recapitulated the transcription defects and morula arrest phenotype in 4KD embryos (Fig. 1d, e; Supplementary information, Fig. S2e–g). Knocking down *Rarg* led to a milder developmental defect, while depletion of *Nr1h3* or *Nr2c2* had negligible effects in preimplantation development (Fig. 1e). To rule out possible off-target effects of *Nr5a2* KD, we attempted to mutate *Nr5a2* via base editing in embryos (Supplementary information, Fig. S3a).^30^ The mutation of *Nr5a2* mRNA and the depletion of protein was confirmed by RNA-seq and immunofluorescence, respectively, at the 8C stage (Fig. 1f, g). Consistently, these mutant embryos largely arrested at the morula stage (Fig. 1h; Supplementary information, Fig. S3b), thus phenocopying the *Nr5a2* KD embryos. Taken together, these results suggest that among the four NR TFs activated upon ZGA, NR5A2 is the dominant factor in regulating early development and transcription.

### NR5A2 is largely dispensable for global ZGA but is required for the 4–8C gene program

**Fig. 2:**
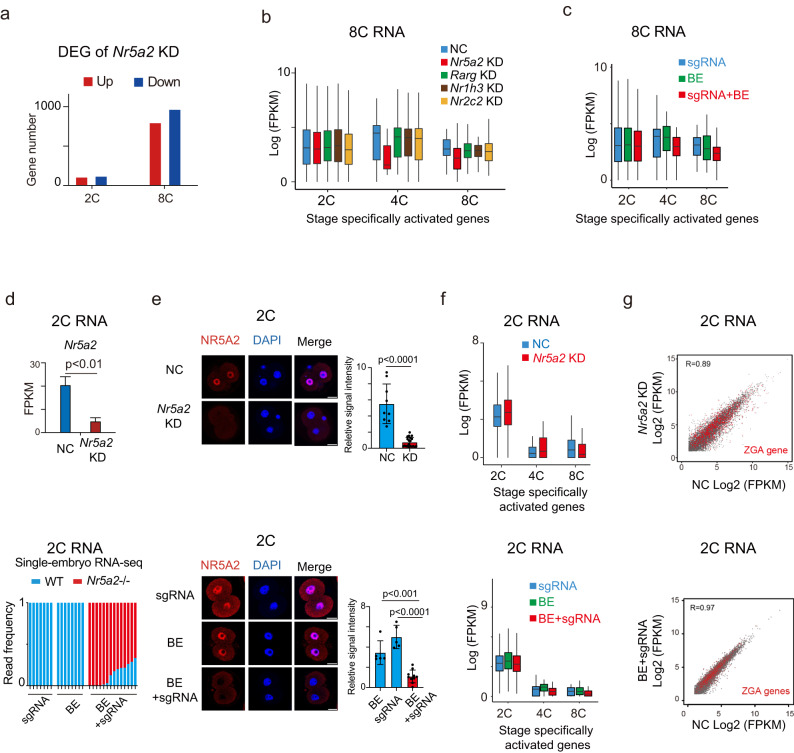
NR5A2 is largely dispensable for global ZGA but is required for 4–8C gene activation. **a** Bar plot showing the numbers of upregulated (red) and downregulated (blue) genes after *Nr5a2* KD in mouse 2C and 8C embryos. **b** Box plots showing the average expression levels of stage-specifically activated genes for NC groups (blue), *Nr5a2* KD groups (red), *Rarg* KD groups (green), *Nr1h3* KD groups (brown), and *Nr2c2* KD groups (yellow) at the 8C stage. **c** Box plots showing the average expression levels of stage-specifically activated genes after injection of sgRNA only (blue), BE mRNA only (green), and both sgRNA and BE mRNA (red) at the 8C stage. **d** Bar charts showing the expression of *Nr5a2* in NC and *Nr5a2* KD group at the 2C stage (left), with *P* values (*t*-test, two-sided) indicated (top). Bar charts show the percentages of WT *Nr5a2* (blue) and base edited *Nr5a2* (red) reads from RNA-seq after injection of *Nr5a2* sgRNA only, BE mRNA only, and both sgRNA and BE mRNA at the 2C stage (bottom). **e** Immunofluorescence of NR5A2 (red) and DAPI (blue) in mouse 2C embryos after *Nr5a2* KD (top, left). Immunofluorescence of NR5A2 (red) and DAPI (blue) in mouse 2C embryos after injection of sgRNA only, BE mRNA only, and both sgRNA and BE mRNA (bottom, left). Scale bars: 20 μm. Quantification of NR5A2 signal intensity (relative to DAPI) in mouse 2C (*n* = 4–7 embryos) and 8C (*n* = 6–8 embryos) embryos and *P v*alues (*t*-test, two-sided) are shown (right). Each dot represents a single blastomere. **f** Box plots showing the average expression levels of stage specifically activated genes for NC groups (blue) and *Nr5a2* KD groups (red) at the 2C stage (top). Box plots show the average expression levels of stage-specifically activated genes after injection of sgRNA only (blue), BE mRNA only (green), and both sgRNA and BE mRNA (red) at the 2C stage (bottom). **g** Scatter plot comparing gene expression of NC group and *Nr5a2* KD group in mouse 2C embryos (top). Scatter plot comparing gene expression of NC group and *Nr5a2* base edited group in mouse 2C embryos (bottom). ZGA genes are colored in red. The Pearson correlation coefficients are also shown.

We then asked how these NR factors including NR5A2 regulate transcription programs in early development. Knocking down *Nr5a2* led to the strongest transcription changes among the 4 NR TFs (Supplmentary information, Fig. S4a), with 976 downregulated and 806 upregulated genes (Fig. 2a; Supplmentary information, Table S3). Furthermore, only *Nr5a2* KD recapitulated the failed activation of 4–8C genes (Fig. 2b). By contrast, the gene dysregulation at the 2C stage was much weaker compared to that at 8C (Fig. 2a, b; Supplementary information, Table S4), with 128 genes downregulated and 115 genes upregulated. A similar observation was made for *Nr5a2* BE KO embryos (Fig. 2c), consistent with the observations that after *Nr5a2* KD or BE KO, embryos progressed beyond 2C normally (Supplementary information, Figs. S2g and S3b). We confirmed the depletion or mutation of *Nr5a2* mRNA and protein at this stage (Fig. 2d, e). Among all examined embryos at the 2C stage (14 embryos), 6 embryos displayed nearly completely mutated *Nr5a2* mRNA reads (96.9%–100%) (Supplementary information, Fig. S4b, the 1st–6th KO embryos), while the remaining 8 embryos exhibited high mutation rates ranging from 66.7% to 87.5% (Supplementary information, Fig. S4b, the 7th–14th KO embryos). The presence of wild-type (WT) transcripts may arise from either unedited alleles or low levels of oocyte-deposited *Nr5a2*. The zygotic genome was globally activated in both *Nr5a2* KD and BE KO embryos (Fig. 2f, g). When using a  defined ZGA gene list (*n* = 1170, Materials and Methods), relatively small and comparable numbers of ZGA genes were upregulated (*n* = 24 and 13, respectively) and downregulated (*n* = 36 and 19, respectively) in KD and BE KO embryos (Fig. 2g; Supplementary information, Fig. S4b and Table S5). In sum, these data suggest that NR5A2 is largely dispensable for the initiation of ZGA but plays a key role in transcriptional regulation especially during the 4–8C stages.

### Genome-wide mapping of NR5A2 chromatin occupancy in mouse early embryos

**Fig. 3:**
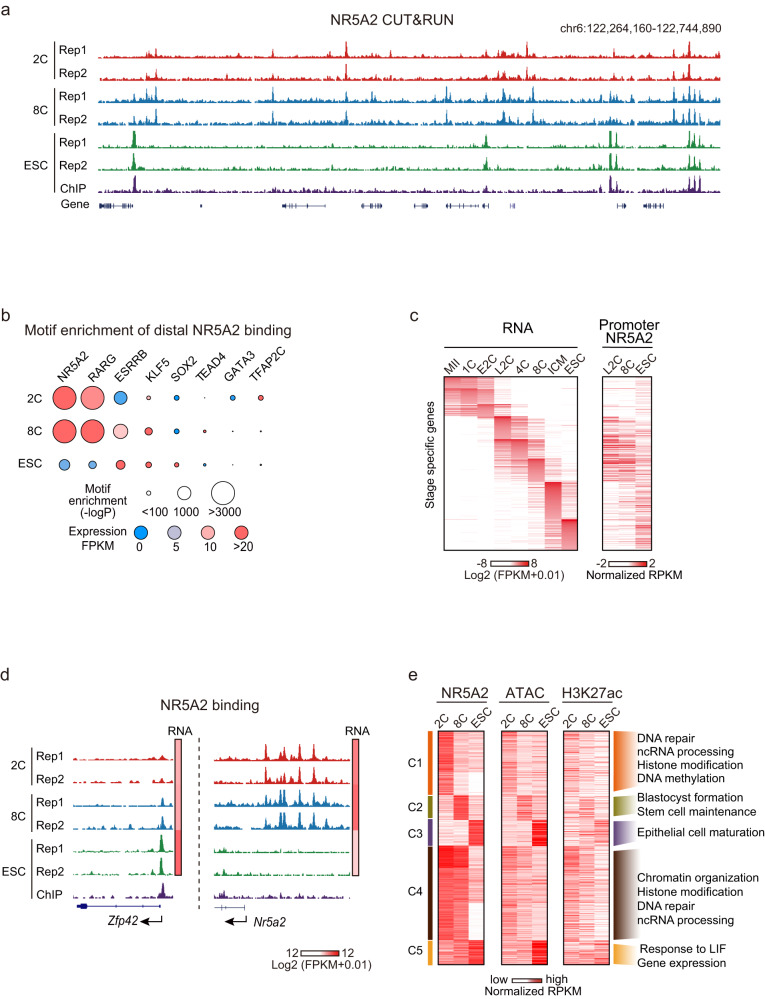
NR5A2 binding dynamics in mouse early embryos. **a** The UCSC browser view showing NR5A2 CUT&RUN signals in 2C embryos, 8C embryos, and mESCs (two biological replicates). NR5A2 ChIP-seq from a reference dataset^24^ is also shown. **b** TF motifs identified from NR5A2 distal binding peaks in 2C embryos, 8C embryos, and mESCs. Sizes of circles indicate –Log*P* value. Expression levels of TFs are color-coded. **c** Heat maps showing stage-specific gene expression based on a reference dataset^17^ and their promoter NR5A2 binding enrichment. **d** The UCSC browser views and heat maps showing NR5A2 enrichment and gene expression of representative genes, respectively. **e** Heatmaps showing the NR5A2 binding, ATAC,^17^ and H3K27ac signals at the stage-specific and shared NR5A2 binding peaks (left). The gene ontology (GO) terms are also shown for different clusters (right).

To further investigate the direct targets of NR5A2 during the mouse early development, we sought to capture its chromatin binding landscape using CUT&RUN.^31^ We identified a sensitive NR5A2 antibody that can detect its binding using as few as 1000 WT mESCs, but not in *Nr5a2* KO mESCs (Supplementary information, Fig. S5a, b). NR5A2 motif was the top 1 enriched motif in its binding peaks (Supplementary information, Fig. S5c), thus validating the data. Next, we performed NR5A2 CUT&RUN in the 2C and 8C embryos, which were highly reproducible (Fig. 3a; Supplementary information, Fig. S5d and Table S6). We could not detect NR5A2 binding in ICM likely due to its low expression (Fig. 1b) and the limited cells we collected. Therefore, we included mESCs for comparison instead. As a strong validation, the NR5A2 motif was again enriched in 2C and 8C binding peaks as the top 1 motif (Fig. 3b; Supplementary information, Table S7). At the 2–8C stages, NR5A2 showed distinct binding compared to that in mESCs and was preferentially enriched at the promoter of 2–8C specifically expressed genes (Fig. 3c; see Fig. 3d for example). In distal regions, NR5A2 also occupied sites specifically marked by H3K27ac and open chromatin,^17^ suggesting that NR5A2 preferentially bound active regulatory regions (Fig. 3e). Stage-specific binding analysis showed that 2C-specific and 2–8C shared NR5A2 distal bindings were enriched near genes involved in housekeeping activities including DNA repair, RNA processing, histone modification, and DNA methylation (Fig. 3e, “C1” and “C4”). Interestingly, 8C-specific NR5A2 binding tended to enrich near genes functioning in blastocyst formation and stem cell maintenance, indicating the possible involvement of NR5A2 in lineage regulation at this stage (Fig. 3e, “C2”). By contrast, mESC-specific binding was found near genes functioning in epithelial cell maturation (Fig. 3e, “C3”). LIF-responding genes also showed higher enrichment of NR5A2 in mESC (Fig. 3e, “C5”). Notably, NR5A2 also bound the putative enhancers near *Nr5a2* itself (Fig. 3d), suggesting a positive feedback regulatory loop. Taken together, these data demonstrate that we successfully captured NR5A2 binding in 2C and 8C embryos.

### NR5A2 targets are preferentially downregulated upon its deficiency

**Fig. 4:**
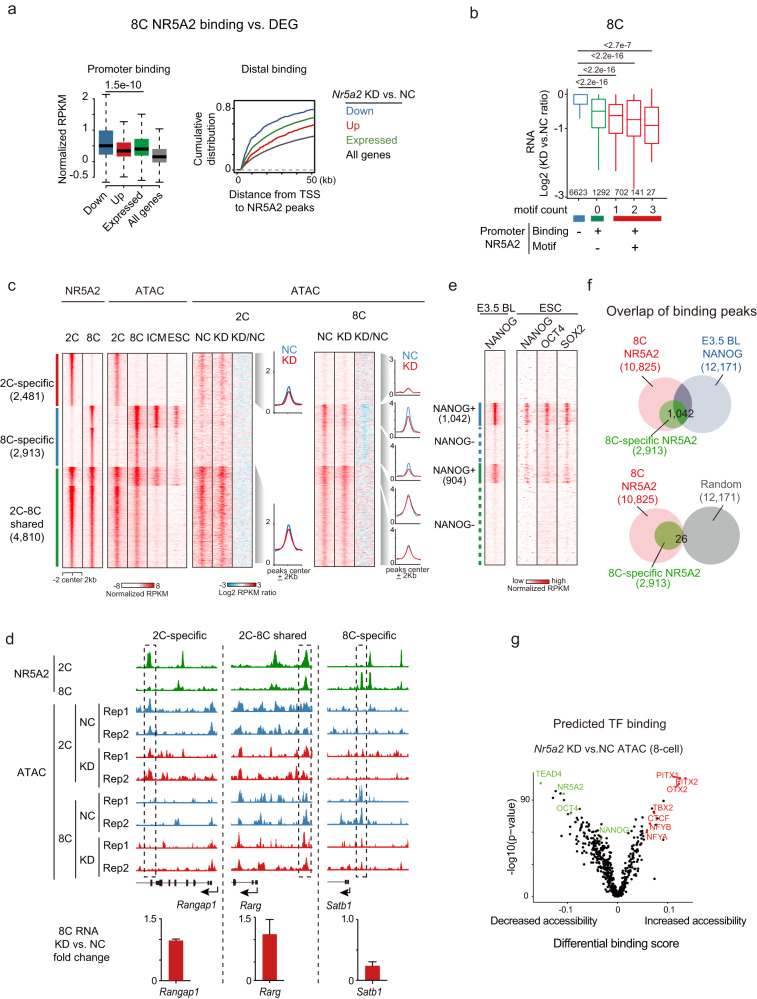
NR5A2 regulates the expression of its binding targets in early development. **a** Box plots showing the average enrichment of NR5A2 binding signals at the promoters (TSS (transcription starting site) ± 2.5 kb) (in WT embryos) of downregulated, upregulated, expressed, and all genes identified in *Nr5a2* KD 8C embryos (left), with *P* values (*t*-test, two-sided) indicated. The cumulative distributions of downregulated, upregulated, expressed, and all genes with defined distances (*x*-axis) between their TSSs and nearest distal 8C NR5A2 binding peaks are shown (right). **b** Box plots showing the fold changes of gene expression in *Nr5a2* KD 8C embryos for all expressed genes based on the NR5A2 binding states and the numbers of motifs at promoters, with *P* values (*t*-test, two-sided) indicated. **c** Heatmap showing the NR5A2 binding, enrichment of ATAC-seq signals in WT reference,^17^ NC, *Nr5a2* KD 2C and 8C embryos. Average plots show enrichment of ATAC-seq signals in NC and *Nr5a2* KD 8C embryos at the 2C-specific, 8C-specific, and 2–8C shared NR5A2 distal binding peaks. **d** The UCSC browser views showing NR5A2 binding, ATAC-seq signals of representative genes in 2C and 8C NC, and *Nr5a2* KD embryos (top). Bar plots shows the expression fold-changes of representative genes between *Nr5a2* KD and control 8C embryos of two biological replicates of RNA-seq (bottom). **e** Heatmap showing NANOG binding in E3.5 blastocyst and NSO binding in mESC at 2C-specific, 8C-specific, and 2–8C shared NR5A2 distal binding peaks. **f** Venn diagrams showing the overlap among 8C NR5A2 binding peaks, 8C-specific NR5A2 binding peaks, and E3.5 blastocyst NANOG peaks (top). Random peaks shuffled from the genome with matched lengths are also shown (bottom). **g** Volcano plot showing the TF footprints in differentially accessible regions in ATAC-seq upon *Nr5a2* KD at the 8C stage. Example footprints in regions with increasing and decreasing accessibilities are colored in red and green, respectively. *P* values for their enrichment are also shown on the *y*-axis.

We then asked whether NR5A2 regulates its binding targets by primarily focusing on the 8C stage when the impact of NR5A2 on transcription was evident. NR5A2 binding was preferentially enriched at the promoters of downregulated but not upregulated genes upon *Nr5a2* KD (Fig. 4a, left), suggesting that it functions primarily as an activator. Consistently, distal NR5A2 binding peaks also preferentially resided near the downregulated genes (Fig. 4a, right). Supporting a direct activation role of NR5A2, genes with NR5A2 binding and more NR5A2 motifs at their promoters were more downregulated in *Nr5a2* KD embryos (Fig. 4b). Among 976 downregulated genes at the 8C stage in *Nr5a2* KD embryos, a significant fraction (27%, *n* = 263, *P* < 0.001) showed NR5A2 binding at promoters. These data demonstrated that NR5A2 directly regulates gene activation at the 8C stage.

To further investigate whether NR5A2 regulates chromatin accessibility at putative enhancers, we performed ATAC-seq in the negative control (NC) and *Nr5a2* KD embryos. We classified distal NR5A2 peaks into 2C-specific, 8C-specific, and 2–8C shared peaks (Fig. 4c). At the 2C stage, chromatin accessibility was globally unaltered (Fig. 4c, “2C ATAC” and Fig. 4d, “*Rangap1*”). The closing of 2C-specific peaks at the 8C stage was unaffected in *Nr5a2* KD 8C embryos. 2–8C shared NR5A2 occupied peaks remained accessible (Fig. 4c, d, “*Rarg*”). However, the opening of 8C-specific NR5A2 peaks was specifically impaired (Fig. 4c, “8C ATAC” and Fig. 4d “*Satb1*”). Among 8C-specific ATAC-seq peaks, 73.9% showed reduced chromatin accessibility upon *Nr5a2* KD. The affected regions were relatively more enriched in distal regions (78.6%) compared to those that were not affected (63.5%). These data indicate that NR5A2 is required for opening 8C-specific putative enhancers but not those opened at the 2C stage (Fig. 4c, d), consistent with NR5A2 being required for 4–8C, but not 2C, global gene activation.

### NR5A2 opens a subset of regions that are bound by pluripotency TFs at later stages

NR5A2 is closely linked to pluripotency regulation.^22,24,25^ Therefore, we asked whether NR5A2 binding at the 8C stage may be related to the future binding of pluripotency factors such as NSO. We analyzed the binding of NANOG in blastocysts^32^ and the binding of NSO in mESC.^33,34^ Intriguingly, 8C-specific, but not 2C-specific NR5A2 binding preferentially enriched for NANOG binding in blastocyst and NSO binding in mESC (Fig. 4e). There was a small group of 2–8C shared ATAC-seq peaks enriched in Nanog binding in the blastocysts (Fig. 4e). About 1042 8C-specific NR5A2 peaks were also occupied by NANOG in blastocysts (Fig. 4f). These regions became less open upon *Nr5a2* KD in the 8C embryos (Fig. 4c, “8C ATAC”). As we could not determine NANOG and OCT4 binding at the 8C embryos due to its low expression (NANOG) and the lack of sensitive antibodies that could be applied to low-input samples, we searched for footprints of TFs within the differentially accessible sites using TOBIAS.^35^ As a validation, the NR5A2 motif showed a significant decrease in ATAC-seq upon *Nr5a2* KD. Consistently, motifs of pluripotency TFs, including NANOG and OCT4, and TEAD4 (a TE regulator) showed decreased ATAC-seq signals upon *Nr5a2* KD (Fig. 4g). In addition, it was reported that ESRRB and NR5A2, which share similar binding motifs and binding sites in mESCs, redundantly regulate NSO binding and pluripotency in mESCs.^27^ Notably, while NR5A2 alone is dispensable for mESC self-renewal,^27^ it plays a much more essential role in embryos likely due to its much higher gene expression (Supplementary information, Fig. S6a). In *Nr5a2/Esrrb* double KO mESCs, the binding of NSO in regions that were co-occupied by ESRRB and NR5A2 (*n* = 2599) was severely disrupted (Supplementary information, Fig. S6b). As a comparison, NSO binding to regions that exhibited weak or no ESRRB/NR5A2 binding was less affected (Supplementary information, Fig. S6b). The dependence of NSO binding on NR5A2/ESRRB in mESCs extended to the regions that were preferentially bound by NR5A2 both in 8C embryos and mESCs, as well as by NANOG in blastocysts (Supplementary information, Fig. S6c). These data raise a possibility that NR5A2 may open a subset of regions at the 8C stage, possibly in anticipation of future binding of pluripotency TFs at later stages.

### NR5A2 regulates early ICM genes and other lineage regulator genes at the 8C stage

**Fig. 5:**
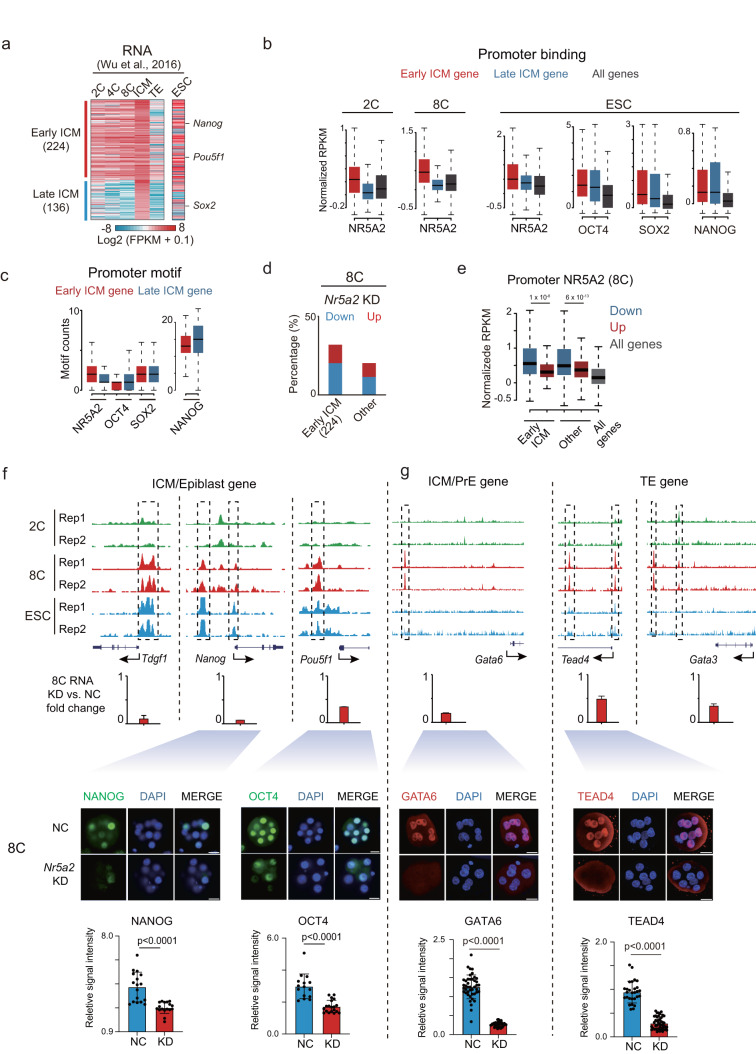
NR5A2 regulates the early ICM, TE and PrE genes at the 8C stage. **a** Heatmap showing the gene expression levels in 2C, 4C, and 8C embryos, E3.5 ICM, TE, and mESC for the early/late ICM genes. Early ICM gene: FPKM ≥ 1 in 8C embryos. Late ICM gene: FPKM < 1 in 8C embryos. **b** Box plots showing the average enrichment of NR5A2 and NSO binding signals at the promoters (TSS ± 2.5 kb) of early or late ICM genes at each stage. **c** Box plots showing the motif numbers of NR5A2 and NSO at the promoters (TSS ± 2.5 kb) of early or late ICM genes. **d** Bar plots showing the percentages of downreuglated and upregulated genes among early, late ICM, or other expressed genes in *Nr5a2* KD 8C embryos. **e** Box plots showing the average enrichment of NR5A2 signals at the promoters (TSS ± 2.5 kb) (in WT embryos) of downregulated or upregulated genes identified in *Nr5a2* KD 8C embryos for early ICM genes and other 8C-expressing genes, with *P* values indicated. All genes were similarly analyzed and are shown as controls. **f**, **g**  The UCSC browser views showing NR5A2 binding of representative ICM/epiblast (**f**) or ICM/PrE and TE genes (**g**) in WT embryos and mESCs (top). Bar plots show expression fold change (*Nr5a2* KD/control) for representative genes in 8C embryos of two biological replicates of RNA-seq (middle). Immunofluorescence of DAPI, NANOG (*n* = 11–12 embryos), OCT4 (*n* = 9–12 embryos), GATA6 (*n* = 13–14 embryos) or TEAD4 (*n* = 12–14 embryos) in mouse 8C embryos after *Nr5a2* KD (scale bars: 20 μm) and quantification of signal intensity (relative to DAPI) as well as *P v*alues (*t*-test, two-sided) are shown. Each dot represents a single blastomere.

We then sought to investigate whether NR5A2 may directly regulate the pluripotency program at the early stage. Globally, we identified 360 genes that were specifically expressed in ICM compared to TE using a reference dataset,^17^ and among them 62% (*n* = 224) were already expressed at the 8C stage (fragments per kilobase of exon per million mapped fragments (FPKM) > 1), which we termed as early ICM genes (Fig. 5a). The rest of them were termed as late ICM genes (Fig. 5a). The expected lineage genes in corresponding classes (Early ICM: *Nanog*, *Pou5f1*; Late ICM: *Sox2*) validated the gene list (Fig. 5a). We then examined the enrichment of NR5A2 binding near the early and late ICM genes. Intriguingly, NR5A2 showed strong binding both at promoters (Fig. 5b) and in distal regions (Supplementary information, Fig. S7a) near early ICM genes, but not near late ICM genes in 2C and 8C embryos. This was also true in mESCs, where both early and late ICM genes were expressed (Fig. 5a). Consistently, the NR5A2 motif was more enriched at the promoter or the nearby regions of early ICM genes compared to late ICM genes (Fig. 5c; Supplementary information, Fig. S7b), suggesting that NR5A2 preferentially regulates early wave of ICM genes. In line with this notion, 45 out of 224 (20%) early ICM genes were downregulated in *Nr5a2* KD 8C embryos, compared to 929 out of 9744 (9.5%) non-early ICM genes expressed at the 8C stage (Fig. 5d). The downregulated, but not the upregulated, early ICM genes were more enriched for NR5A2 binding at their promoters or in their neighbor distal regions (Fig. 5e; Supplementary information, Fig. S7c), indicating that they are part of direct targets of NR5A2. NR5A2 peaks near the early ICM genes also became less accessible upon *Nr5a2* KD (Supplementary information, Fig. S7d). For example, it bound pluripotency genes *Oct4*, *Nanog*, and *Tdgf1* which were all downregulated in *Nr5a2* KD 8C embryos (Fig. 5f). NANOG and OCT4 proteins were both decreased in *Nr5a2* KD and, to a lesser extent, *Nr5a2* KO 8C embryos (Fig. 5f; Supplementary information, Fig. S8a). Moreover, 8C-specific NR5A2 binding peaks with future NANOG binding were preferentially present near early ICM genes, which also exhibited decreased accessibility upon *Nr5a2* KD (Fig. 4c; Supplementary information, Fig. S8b), as exemplified in *Id3* (Supplementary information, Fig. S8c). Early ICM genes tended to be downregulated rather than upregulated upon *Nr5a2* KD at the 8C stage, an effect that was more evident for those with NR5A2 binding than those not bound by NR5A2 (Supplementary information, Fig. S8b). Together, these results suggest that NR5A2 directly binds and promotes transcription of early ICM genes at the 8C stage including a subset of key pluripotency genes. Of note, a few key PrE regulator genes *Gata6* and *Fgfr1/2*, and TE regulator genes, including *Tead4, Klf5, Tfap2c* and *Gata3* were also bound by NR5A2 and downregulated in *Nr5a2* KD 8C embryos (Fig. 5g), suggesting that NR5A2 may also regulate TE and PrE lineages.

Interestingly, NR5A2 alone is dispensable for mESC pluripotency and self-renewal.^22^ Consistently, *Nr5a2* KO affected limited ICM-specific genes in mESC, unlike that for SOX2 (Supplementary information, Fig. S8d), which echoed the absence of NR5A2 motif in ATAC-seq peaks and its downregulated expression in ICM and mESC (Fig. 1a, b). We reasoned that the pluripotency transcription network is likely taken over by the pluripotency TFs NSO in ICM and mESC based on the motif analysis from ATAC-seq data (Fig. 1a). Indeed, the motifs of NSO were more enriched near late ICM genes compared to early ICM genes (Fig. 5c; Supplementary information, Fig. S7b). Interestingly, while these factors clearly bound late ICM genes in blastocyst (for NANOG) or ESCs (NSO), they also bound early ICM genes as well (Fig. 5b). To ask to what extent pluripotency TFs directly regulate the early and late ICM genes, we fused FKBP12^F36V^ sequence to the C-terminus of the endogenous SOX2 protein in 2i mESC so that SOX2 protein can be rapidly degraded by the addition of dTAG.^36^ We then performed RNA-seq at 24 h and 48 h after SOX2 degradation (Supplementary information, Fig. S8d). The result showed that 15.5% early and 14.6% late ICM genes were significantly downregulated upon SOX2 depletion within 1 day, and the number increased to 23.5% early and 32.3% late ICM genes within 2 days, which contrasted a negligible role of NR5A2 in transcription in mESC (Supplementary information, Fig. S8d). Together, these data indicate that while NR5A2 preferentially regulates early ICM genes, NSO regulate both early and late ICM genes. NR5A2 not only activates the transcription of pluripotency factor genes like *Oct4* and *Nanog*, but also opens a subset of sites that will be bound by NSO at the later stage.

### NR5A2 predominantly occupies the B1 repeats in the 2C and 8C embryos

**Fig. 6:**
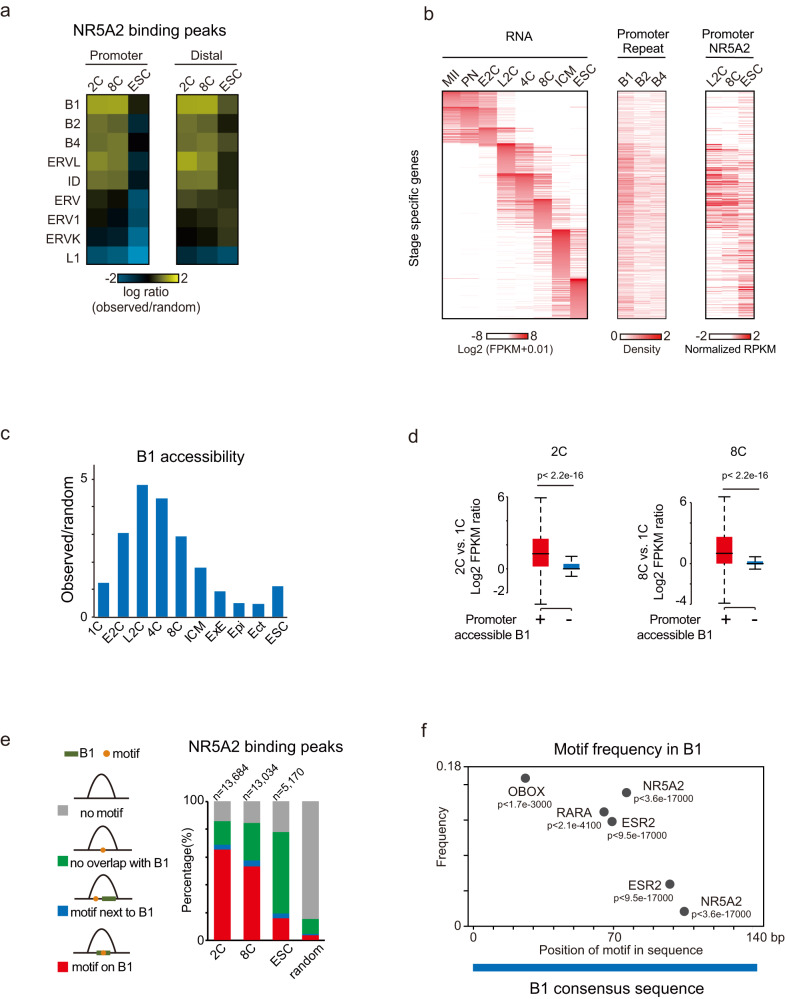
NR5A2 binds B1 repeats at the 2–8C stage. **a** Heat maps showing enrichment (observed number/expected number from randomized peaks) of each repeat family in promoter and distal NR5A2 binding peaks. **b** Heat maps showing the gene expression, repeat density, and NR5A2 binding enrichment at the promoters of stage-specific genes. E2C, early 2-cell; L2C, late 2-cell. **c** Bar plots showing the enrichment of accessible B1 repeats based on ATAC-seq and DHS-seq data relative to the background (observed number/expected number) in early embryos. ExE, extraembryonic ectoderm; Epi, epiblast; Ect, ectoderm. **d** Box plots showing expression activation levels (late 2C/1C (left) and 8C/1C (right)) for genes near accessible or inaccessible B1 at the 2C and 8C stages, with *P* values indicated. The error bars denote the standard deviations of two biological replicates of RNA-seq. **e** Bar charts showing percentages of NR5A2 binding peaks at each stage in four types: motif in B1 (red), motif next to B1 (blue), motif non-overlapping with B1 (green), and no motif (gray). **f** TF motif frequencies and relative positions in the B1 consensus sequence are shown, with *P* values indicated.

One interesting question is why NR5A2 binds many early-stage genes in embryos but not in mESCs. Repetitive elements are known to harbor the repertoire of TF binding sites.^37^ Several classes of transposable elements such as B1, B2, and MERVL are specifically activated in mouse preimplantation embryos.^17,38–41^ Interestingly, NR5A2 binding sites at the 2C and 8C stages, both for those at promoters and distal regions, overwhelmingly overlapped with the short-interspersed element (SINE) family, especially with the B1 repeats (Fig. 6a; Supplementary information, Fig. S9a). For example, 84.8% and 73.3% of NR5A2 binding peaks contained B1 at the 2C and 8C stages, respectively, compared to 18.9% of random peaks (Supplementary information, Fig. S9a). B1 was abundantly expressed^42^ and was indicated to play critical roles in mouse early embryogenesis.^40^ B1 was preferentially present near 2–8C gene promoters, consistent with previous work,^43^ and correlated with NR5A2 binding at the 2C and 8C stages (Fig. 6b). B1 became widely accessible from the early 2C stage until the 8C stage based on ATAC-seq^17^ and DHS-seq^44^ data (Fig. 6c). Genes with accessible B1 at the promoters in the late 2C and 8C stages were more likely to be activated compared to genes with inaccessible B1 at the promoters (Fig. 6d). The accessible B1 at the 2C stage was even more enriched for the NR5A2 motif (66.4%) compared to all B1 elements (52.5%). To further investigate the association of NR5A2 binding and B1 repeats, we classified the NR5A2 peaks into 4 groups based on the presence or absence of its motif and the B1 repeats, and whether the motif, if present, falls into B1 (Fig. 6e). At the 2C stage, the majority of NR5A2 binding peaks (64.9%) tended to contain B1 with the NR5A2 motif (Fig. 6e). This percentage then slightly decreased in 8C embryos (52.7%), and dropped abruptly in mESCs (15.6%). By contrast, NR5A2 preferentially bound its motif outside of B1 in mESCs (58.3% of all binding peaks) (Fig. 6e), suggesting that the enriched binding of NR5A2 to B1 elements is specific for early embryos.

The data above raised two possibilities: the binding of NR5A2 may help open B1; alternatively, B1 may be pre-opened by other factors that facilitate the binding of NR5A2. The latter was supported by the fact that B1 became accessible starting from the early 2C stage (Fig. 6c), when *Nr5a2* expression was still low (Fig. 1b). Notably, the consensus sequence of B1 harbored motifs not only for NR5A2, but also for other TFs such as OBOX, RARa, and ESR (Fig. 6f). Similarly, other transposable elements such as B2 and B4 also possessed a set of other TF motifs (Supplementary information, Fig. S9b). Of note, we recently identified OBOX as a key regulator of mouse ZGA and early embryogenesis, which preferentially binds B1/B2/B4 repetitive elements and opens promoters and enhancers near ZGA genes.^45^ OBOX also binds and activates *Nr5a2*.^45^ Future studies are warranted to investigate whether OBOX or other TFs may open these regions to facilitate the binding of NR5A2 to exert gene activation of downstream targets.

## Discussion

**Fig. 7:**
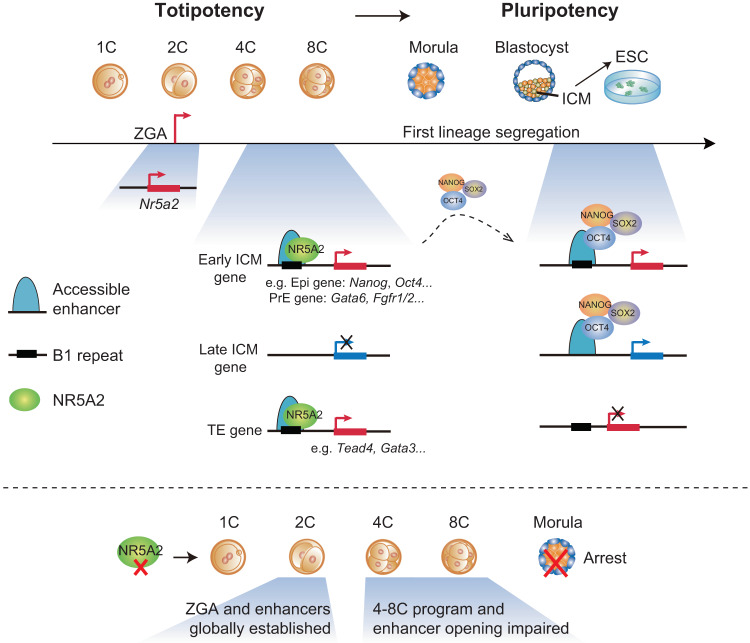
NR5A2 connects ZGA to the first lineage specification in early development. During mouse early development, NR5A2, a NR TF highly induced during ZGA, predominantly binds B1 repeats. NR5A2 is not required for global ZGA and enhancer opening in 2C embryos, but critically regulates 4–8C transcription (mid-preimplantation gene activation) and enhancer opening in 8C embryos. In 8C embryos, its targets include not only key pluripotency genes such as *Pou5f1* and *Nanog*, but also PrE genes such as *Gata6*,  and key TE genes such as *Tead4*, *Gata3*. For early ICM genes, NR5A2 opens regulatory regions at the 8C stage, which likely also provides binding sites for NSO at late stages (blastocyst and mESC). NSO bind and regulate both early and late ICM genes in mESCs. Therefore, NR5A2 functions in totipotent embryos to bridge ZGA to the first lineage specification.

TFs play essential roles in gene regulation and animal development. However, how TFs guide mammalian preimplantation development remains poorly understood. Here, through functional screening of TFs that were highly expressed after ZGA and showed motif enrichment in accessible chromatin, we identified NR5A2 as a key regulator of mouse early development and transcription program. The KD or KO of *Nr5a2* from the 1C stage led to preimplantation development defects, concomitant with the 4–8C gene program impaired. Using CUT&RUN, we further determined possible direct targets of NR5A2 in early embryos. We found that NR5A2 connects genome activation to the first lineage segregation not only by directly activating the expression of a panel of key pluripotency genes (i.e., *Nanog*, *Pou5f1*, and *Tdgf1*) at the 8C stage, but also opening a subset of sites that will be bound by NSO at later stages in blastocysts and mESCs (Fig. 7). In addition to pluripotency genes, NR5A2 also binds and regulates key PrE genes such as *Gata6*, and key TE genes such as *Tead4*, *Gata3* (Fig. 7). Intriguingly, NR5A2 bound extensively to the B1 elements at the 2–8C stages, but less so in mESCs. This coincides with the widespread opening of B1 in mouse embryos. These data demonstrate a critical role of NR5A2 in mouse early development by connecting ZGA to lineage segregation.

Despite a vast amount of knowledge on the core pluripotency TFs and their targets, their upstream TF regulators in early embryos are still poorly characterized. The loss of *Nanog*, *Pou5f1*, or *Sox2* did not affect the formation of blastocysts in mice,^12,13,46^ suggesting that at least the initiation and commitment of the first lineage segregation does not strictly require these factors. Identification of upstream TFs is in part hindered by the limited research materials from early embryos and the lack of a cell line in vitro that can well recapitulate early embryo development, especially during the period of ZGA to morula. On the other hand, ATAC-seq and related methods coupled with RNA-seq provide a powerful tool to identify possible TF regulator candidates. As a testimony of this approach, NR5A2 represents one of the earliest TFs identified to date that exert functions on gene programs in early embryos, at least starting from the 4C stage. Importantly, NR5A2 is required for the proper expression of a number of key pluripotency genes including *Pou5f1* and *Nanog* at the 8C stage. This is consistent with the previous finding that NR5A2 could directly activate *Nanog* during iPSC reprograming.^24^ On the other hand, NR5A2 opens a subset of sites that are bound by NSO at a later stage (blastocyst) or mESC. Future studies are warranted to ultimately test whether the deficiency of NR5A2 affects the binding of NANOG or OCT4 in 8C embryos. Interestingly, NR5A2 preferentially regulates early ICM genes, but not late ICM genes in the 8C embryos and ESCs, in line with the fact that *Nr5a2* is downregulated in blastocyst (Fig. 1b) and it alone is dispensable for mESC self-renewal and pluripotency.^26,27^ A plausible possibility for the diminished role of NR5A2 for ICM genes (including early ICM genes) at later stages is that when pluripotency core TFs are fully expressed, they form interconnected regulatory circuitry^11,47^ and take over the pluripotency regulatory network to regulate both early and late ICM genes, without requiring the continuous activation by NR5A2.

Of note, the *Nr5a2* KD and KO phenotypes in this study were more severe than those reported in previous studies.^22,48^ Given that these studies targeted different regions of *Nr5a2* for gene KO, it remained to be determined if the discrepancy may arise from these differences. Of note, a recent study reported a requirement of maternal NR5A2 in mouse ZGA and development beyond the 2C stage, using an NR5A2 chemical inhibitor and NR5A2 Trim-away followed by RNA-FISH.^49^ Only a moderate effect on ZGA was found using *Nr5a2* KD.^49^ By contrast, *Nr5a2* KD and KO starting from the 1C stage in our experiments did not have a major impact on ZGA and 2C development. Despite the low expression of maternal *Nr5a2* (Fig. 1b), we could not assess its potential role in our study. However, our data are fully consistent with a recent preprint showing that ZGA largely initiated normally in *Nr5a2* maternal–zygotic KO embryos which were subsequently arrested at the morula stage, and maternal NR5A2 was dispensable for embryonic development.^50^ These discrepancies likely arise from different approaches used in these studies.

Our NR5A2 CUT&RUN data in the embryos provide strong functional support for previously identified putative regulatory elements marked by ATAC-seq and histone acetylation,^17^ as they interact with NR5A2 and the nearby genes were preferentially downregulated upon *Nr5a2* KD. Interestingly, NR5A2 pervasively occupied motifs embedded in accessible B1 elements in 2C and 8C embryos. Owing to the global epigenetic resetting after fertilization, the chromatin landscape of the mouse early embryo is extensively shaped by transiently expressed transposable elements, such as B1, B2, B4, and MERVL.^17^ Once regarded as the junk of the genome,^51,52^ transposable elements are now considered as key players in gene regulation through *cis*- or *trans*-regulatory mechanisms.^37^ These functions are in part mediated by their embedded TF motifs.^53,54^ For example, the B2 repeats possess the CTCF motif that can actively wire chromatin organization.^55,56^ In mouse embryos, the KD of B1 induced 2C arrest,^57^ although the underlying mechanisms remain unclear. Given that B1 elements are already accessible prior to major ZGA^2^ (Fig. 6c) and NR5A2 is strongly induced after ZGA (late 2C stage) (Fig. 1b), we favor the possibility that B1, at least at the 2C stage, may be opened by other TFs at least, which further allows the binding of NR5A2 that subsequently triggers downstream events including the activation of pluripotency genes. This may also explain why *Nr5a2* KD has a limited impact on ZGA. We nevertheless cannot rule out the possibility that NR5A2, once expressed, may in turn participate in opening these elements. B1 activation likely requires multiple factors, as B1 elements are rarely bound by NR5A2 and are mostly inaccessible in mESCs. The relationship between B1 activities and the binding of NR5A2 as well as other TFs remains to be further explored. In sum, we identified NR5A2 as a critical TF regulator for the 4–8C stage-specific transcription in totipotent mouse embryos, thus filling a major gap of transcription circuitry in early development that connects ZGA to lineage segregation. The TF-regulome maps presented here also pave the ways for future studies to decode transcription circuitry underlying early development and cell fate decisions when life begins. Finally, given that *Nr5a2* expression is low in human embryos unlike its counterpart in mouse embryos^58,59^ (Supplementary information, Fig. S10a), it would be highly interesting to investigate whether other nuclear receptor TFs may execute similar functions in human early development.

## Materials and methods

### Data reporting

No statistical methods were used to predetermine sample size. The experiments were not randomized and the investigators were not blinded to allocation during outcome assessment.

### Animal maintenance

WT C57BL/6J strain mice were purchased from Vital River (Beijing, China). PWK/PhJ mice were originally purchased from Jackson Laboratory and raised at Tsinghua Animal Center. Mice were maintained under SPF conditions with a 12–12 h light-dark cycle in a 20–22 °C environment. All animals were taken care of according to the guidelines of the Institutional Animal Care and Use Committee (IACUC) of Tsinghua University, Beijing, China.

### Early embryo collection

To obtain mouse preimplantation embryos, 5–6-week-old C57BL/6N female mice were intraperitoneally injected with pregnant mare’s serum gonadotropin (PMSG; 5 IU) and human chorionic gonadotrophin (hCG; 5 IU) 44–48 h after PMSG injection. After mating with PWK/PhJ males, preimplantation embryos were collected in M2 medium at the following time points after hCG injection: 43–45 h (late 2C) and 68–70 h (8C).

### Cell culture and *Nr5a2* KO mESC generation

mESC were cultured on 0.1% gelatin pre-coated dish in DMEM (Gibco, 11995-065) containing 15% FBS (Hyclone, SH30396.03), LIF (Millipore, GSE1107), penicillin/streptomycin (Millipore, TMS-AB2-C), GlutaMAX (Gibco, 35050-061), nucleosides (Millipore, ES-008-D), β-mercaptoethanol (Gibco, 21985-023), and non-essential amino acids (Gibco, 25-025-CIR). To target the *Nr5a2* gene, four sgRNA sequences (GTACTATAAGCACGTGAACG, TTCTTCGGCTACCCGAGATC, GCTAAGAATGTCTGCTAGTT, GAAGCCACTCTCTTAACATC) were individually cloned into a pX330 plasmid (Addgene, 42230) and were co-transfected into the R1 mESCs with Lipofectamine 3000 (ThermoFisher Scientific). Two to three days after transfection, cells were manually sorted into a gelatinized 96-well plate for single-clone selection. The obtained clones were genotyped by PCR and validated by Sanger sequencing. The homozygous *Nr5a2* KO cell lines were further validated by western blot analysis for NR5A2.

### Immunostaining and quantification

All steps were performed at room temperature. Mouse embryos were fixed with 4% paraformaldehyde^37^ (Sigma-Aldrich, P6148) for 30 min and then permeabilized with 0.5% Triton X-100 in PBS for 30 min. The samples were blocked with 1% BSA for 1 h and incubated with primary antibodies (NR5A2: R&D, P-H2325-00; NANOG: CST, 8822T; OCT4: CST, 5677; TEAD4: Abcam, ab58310; GATA6: R&D, AF1700) for 1 h. The primary antibody was washed out with PBST (0.1% Triton X-100 in PBS) and then incubated with the secondary antibody and Hoechst 33342 for 30 min. The samples were washed with PBST three times. All immunofluorescence images were taken by LSM880 confocal microscope system (Zeiss) and were analyzed using ImageJ. The midsection of each nucleus was identified by Hoechst 33342 staining and determined by the maximal area using a custom Python script. The protein levels of midsections were quantified and normalized to the Hoechst 33342 signals for each experiment.

### In vitro transcription and base editor

The sgRNA (ggtccgatcgcatggggaac) was cloned into pX330 vectors (Addgene, 42230). The primers ttaatacgactcactatagggtccgatcgcatggggaac and aaaagcaccggactcggtgcc were used to obtain linearized sgRNA with *T7* promoter. The pCMV-hA3A-eBE-Y130F (Addgene, 113423) plasmids were linearized. The linearized sgRNA and eBE plasmids were then transcribed with *T7* mMESSAGE Kit (Invitrogen, AM1334) following the manufacturer’s instructions. mRNAs were recovered by RNAClean XP beads (Beckman, A63987). 100 ng/μL sgRNA and BE mRNA were used. All injections were performed with an Eppendorf Transferman NK2 micromanipulator.

### Genotyping

Genomic DNA was lysed from single edited blastocyst embryos for PCR genotyping and subjected to targeted Sanger sequencing.

### NR TF KD

To KD four NR family members *Nr5a2, Rarg*, *Nr1h3*, and *Nr2c2*, three siRNAs were designed for each gene. The siRNA of NC is UAAGGCUAUGAAGAGAUACTT. The siRNAs of *Nr5a2* are CCUCUGCAAUUCAGAACAUTT, GCUCACCUGAGUCAAUGAUTT, GGAGUGAGCUCUUGAUUCUTGT. The siRNAs of *Rarg* are GCCAUGCUUUGUAUGCAAUTT, GGAAGCUGUAGAGGAACGAUTT, CUUGUCUGGACAUCCUAAUTT. The siRNAs of *Nr1h3* are CCAUUCAGAGCAAGUGUUUTT, GGCUGCAACACACAUAUGUTT, GUGCAGGAGGAUUGUUGACUTT. The siRNAs of *Nr2c2* are GCUCAUGAGCUCCAACAUATT, GCCAGAGUACCUCAAUGUATT, GCAAAUGUAGUGACCUCUUTT. For microinjection in each KD group, 1.67 μM of each siRNA was used. For the NC group, 20 μM of siRNA was used. siRNAs were ordered from GenePharma.

### RNA-seq library preparation and sequencing

All RNA-seq libraries were generated following the Smart-seq2 protocol as described previously.^60^ The zona pellucida was gently removed by treatment with Tyrode’s solution (Sigma-Aldrich, T1788). 5–10 embryos per sample were washed three times in M2 medium and then lysed in 2 μL lysis buffer containing RNase inhibitor.

### CUT&RUN library generation and sequencing

CUT&RUN was conducted following the published protocol.^61^ ESCs or embryos with zona pellucida removed were transferred into a 0.2 mL conventional, non-low-binding PCR tube (Axygen). The samples were then resuspended by 60 μL washing buffer (20 mM HEPES-KOH, pH 7.5, 150 mM NaCl, 0.5 mM Spermidine, and Roche complete protease inhibitor). 10–20 μL Concanavalin-coated magnetic beads (Polyscience, 86057) for each sample were gently washed, resuspended by binding buffer (20 mM HEPES-KOH, pH 7.9, 10 mM KCl, 1 mM CaCl_2_, 1 mM MnCl_2_) and carefully added to the samples. The samples were then incubated at 23 °C for 10 min on Thermomixer (Eppendorf) at 400 rpm. The samples were then held at the magnetic stand to carefully aspirate buffer and were resuspended by 60 μL antibody buffer (washing buffer plus 0.01% digitonin and 2 mM EDTA, pH 8.0) with an NR5A2 antibody (R&D, P-H2325-00) diluted at a ratio of 1:50. The samples were then incubated at 4 °C on Thermomixer for 3 h at 400 rpm. After that, the samples were held at the magnetic stand and then resuspended by the secondary antibody to a final concentration of 1:100 at 4 °C on Thermomixer for 1 h at 400 rpm. The samples were then placed in the tube on the magnet stand to remove the liquid and added with 75 μL dig washing buffer with pA-MNase (to a final concentration of 700 ng/mL), and incubated at 4 °C on Thermomixer for 1 h at 400 rpm. The samples were washed with 200 μL dig washing buffer twice at the magnetic stand. The samples were resuspended by 100 μL dig washing buffer and balanced on ice for 2 min. Targeted digestion was performed on ice by adding 2 μL 100 mM CaCl_2_ for 30 min, and reaction was stopped by adding 100 μL 2× stop buffer (340 mM NaCl, 20 mM EDTA, pH 8.0, 4 mM EGTA, pH 8.0, 50 μg/mL RNase A, 100 μg/mL glycogen) and fully vortexed. The samples were then incubated at 37 °C for 30–45 min for fragment release. The supernatants were purified by phenol-chloroform followed by ethanol purification.

Purified DNA was subjected to Tru-seq library construction using NEBNext Ultra II DNA Library Prep Kit for Illumina (NEB, E7645S). The DNA was resuspended in 25 μL ddH_2_O, followed by end-repair/A-tailing with 3.5 μL End Prep buffer and 1.5 μL enzyme mix according to manufacture’s instructions. The ligation reaction was then performed by adding diluted 1.25 μL 0.25 μM adapters (NEB Multiplex Oligos for Illumina, E7335S), 15 μL Ligation master mix, and 0.5 μL Ligation enhancer, at 4 °C overnight or 20 °C for 2 h. The ligation reaction was treated with 1.5 μL USERTM enzyme according to the instruction and was purified by 1.4× AMPure beads. The PCR was performed by adding 25 μL 2× KAPA HiFi HotStart Ready Mix (KAPA Biosystems, KM2602) with primers of NEB Oligos kit, with the program of 98 °C for 45 s, (98 °C for 15 s and 60 °C for 10 s) with 16 cycles and 72 °C for 1 min. The final libraries were purified by 1.4× AMPure beads and subjected to next-generation.

### ATAC-seq library preparation and sequencing

The miniATAC-seq procedure was performed as previously described.^62^ Briefly, cells were transferred into 6 μL of lysis buffer (10 mM Tris-HCl, pH 7.4, 10 mM NaCl, 3 mM MgCl_2_, and 0.02% digitonin, 0.1% NP-40) on ice for 10 min. The ATAC reaction was performed by adding 4 μL ddH_2_O, 4 μL 5× TTBL, and 5 μL TTE mix V5 (Vazyme Biotech Co. Ltd, TD502) at 37 °C for 30 min and then stopped by adding 5 μL of 5× TS stop buffer at room temperature for 5 min. DNA was extracted by phenol-chloroform and ethanol purification after adding 40 ng of carrier RNA and 103 μL of Tris-EDTA. Then, DNA was dissolved in 29 μL ddH_2_O and PCR-amplified with 10 μL of index (Vazyme Biotech Co. Ltd, TD202), 10 μL 5× TAB, and 1 μL TAE (Vazyme Biotech Co. Ltd, TD502) with the program of 72 °C for 3 min, 98 °C for 30 s, (98 °C for 15 s, 60 °C for 30 s, and 72 °C for 3 min) with 16 cycles, and 72 °C for 5 min. The amplified DNA was size-selected using AMPure Beads for 200–800-bp DNA fragments. All libraries were sequenced by Illumina NovaSeq or XTen platform accordingly.

### Western blot analysis

The samples were lysed with 2× Tris-glycine-SDS sample buffer including 1× Roche complete protease inhibitor. Samples were heated at 99 °C for 5 min and loaded on a BioRad 4%–15% gradient Tris-glycine gel, then transferred to low fluorescence PDVF membrane using a BioRad System. Blots were briefly rinsed in TBS, blocked in 5% non-fat milk in TBST for 1 h, and incubated overnight at 4 °C with a primary antibody (NR5A2, R&D, P-H2325-00, 1:1000). Blots were washed in TBST, incubated for 1 h at room temperature with peroxidase-conjugated anti-rabbit or anti-mouse IgG antibody (Jackson ImmunoResearch) diluted at 1:10,000 in TBST with 1% non-fat milk, and then washed in TBST. The signal was detected with SuperSignalTM West Femto Maximum Sensitivity Substrate (ThermoFisher Scientific) and imaged using a BioRad ChemiDocTM Touch Imaging system.

### Data analysis

#### RNA-seq data processing

Paired-end RNA-seq reads were trimmed and mapped to the mm9 genome by TopHat v2.1.1.^63^ Cufflinks v2.2.1^63^ was used to calculate the FPKM per gene based on mm9 refFlat from the UCSC genome annotation database.^64^ Differential expression analysis was performed with R package edgeR. Genes with FDR < 0.01 and log_2_(fold change) ≥ 2 were considered as differentially expressed.

#### Identification of stage-specific genes and ZGA genes

ZGA genes and stage-specific genes were defined based on the reference RNA-seq data using staged mouse embryos dissected in vivo. ZGA genes were defined as those not expressed or lowly expressed in FGO and M II oocytes (FPKM < 5) but become upregulated (FPKM > 5, at least 3-fold upregulation) in either E2C embryos or L2C embryos. Genes that are expressed in oocytes (FPKM > 5) but are highly upregulated at the L2C stage (over 5-fold upregulation) were also included in major ZGA genes (*n* = 99). No such genes exist for minor ZGA genes. Maternal genes were defined as those that are expressed in M II or FGO oocytes (FPKM > 5) but become downregulated (at least 3-fold) at the L2C stage. Genes specifically activated at each stage during early development were defined using more strict criteria to ensure their stage specificity. These genes are activated at a defined stage (FPKM > 5) but stay silenced at all preceding stages from FGO (FPKM < 1).

#### CUT&RUN, ChIP-seq, and ATAC-seq data processing and peak calling

The paired-end reads were aligned with the parameters: -t –q –N 1 –L 25 –X 1000 -no- mixed -no-discordant by Bowtie2 (version 2.2.2).^65^ All unmapped reads, non-uniquely mapped reads, and PCR duplicates were removed. For downstream analysis, we normalized the read counts by computing the numbers of reads per kilobase of bin per million of reads sequenced (RPKM) for 100-bp bins of the genome. To minimize the batch and cell type variation, RPKM values across the whole genome were further Z-score normalized. To visualize the CUT&RUN signals in the UCSC genome browser, we generated the RPKM values on a 100-bp-window base. Peaks were called using MACS v1.4.2^66^ with the parameters nolambda –nomodel. Promoters were defined as ±2.5 kb around the TSS. Peaks at least 2.5 kb away from TSSs were defined as distal peaks by BEDTools v2.29.0.^67^

#### Binding site feature annotation and motif analyses

The findMotifsGenome.pl script from HOMER program^68^ was used to identify the enriched motifs at enhancers. AnnotatePeaks.pl was then used to identify specific peaks that contain certain motifs. Motif densities at 10-bp resolution within 500 bp around peak center were analyzed using the annotatePeaks.pl with the parameters: annotatePeaks.pl mm9 -size -500,500 -hist 10.

#### GO analysis

The DAVID web-tool was employed to identify the GO terms using databases including Molecular Functions, Biological Functions and Cellular Components.^69^ The functional enrichment for genes that are near stage-specific distal peaks was analyzed using the GREAT tool by default settings.^70^

#### Identification of stage-specific NR5A2 peaks

In brief, distal peaks from all stages were pooled, and average Z-score normalized RPKM scores were calculated in these regions. MAnorm2^71^ was used to identify differential peaks in any combinations of pairs of all examined stages. Peaks were further selected by the following criteria: the distal peak had high enrichment at this stage (normalized RPKM > 1) and positive enrichment (normalized RPKM > 0) was observed at no more than two additional stages.

#### The comparison between NR5A2 peaks and repetitive elements

To identify the enrichment of repetitive elements in promoter or distal NR5A2 peaks, the NR5A2 peaks were compared with the locations of annotated repeats (RepeatMasker) downloaded from the UCSC genome browser. As repeats of different classes vary greatly in numbers, a random set of peaks with identical lengths of CUT&RUN peaks were used for the same analysis as a control. The number of observed peaks that overlap with repeats were compared to the number of random peaks that overlap with repeats, and a log ratio value (log_2_) was generated as the “observed/expected” enrichment.

#### Enhancer vs gene expression analysis

To identify the potential targeted genes for each putative enhancer (distal peaks), the nearest distal peaks from the TSS of the gene (within 50 kb) were assigned to this gene as its putative enhancers.

### Supplementary information

Supplementary Fig. S1

Supplementary Fig. S2

Supplementary Fig. S3

Supplementary Fig. S4

Supplementary Fig. S5

Supplementary Fig. S6

Supplementary Fig. S7

Supplementary Fig. S8

Supplementary Fig. S9

Supplementary Fig. S10

Supplementary Table S1

Supplementary Table S2

Supplementary Table S3

Supplementary Table S4

Supplementary Table S5

Supplementary Table S6

Supplementary Table S7

## Data Availability

The raw sequencing data and processed data of this paper have been deposited in the Gene Expression Omnibus (GEO) database under the accession number: GSE229740. Accession codes of the published data in GEO used in this study are as follows: RNA-seq and ATAC-seq of the mouse early embryos, GSE66390; NR5A2 ChIP of mESC, GSE19019.
