# Supplementary material for: NR5A2 connects zygotic genome activation to the first lineage segregation in totipotent embryos
Source: Cell Res. 2023 Nov 7;33(12):952–66. doi: 10.1038/s41422-023-00887-z (PMC10709309; doi:10.1038/s41422-023-00887-z)
Supplement: Supplementary file 1 — Supplementary Fig. S1 [file 41422_2023_887_MOESM1_ESM.pdf]

Figure S1

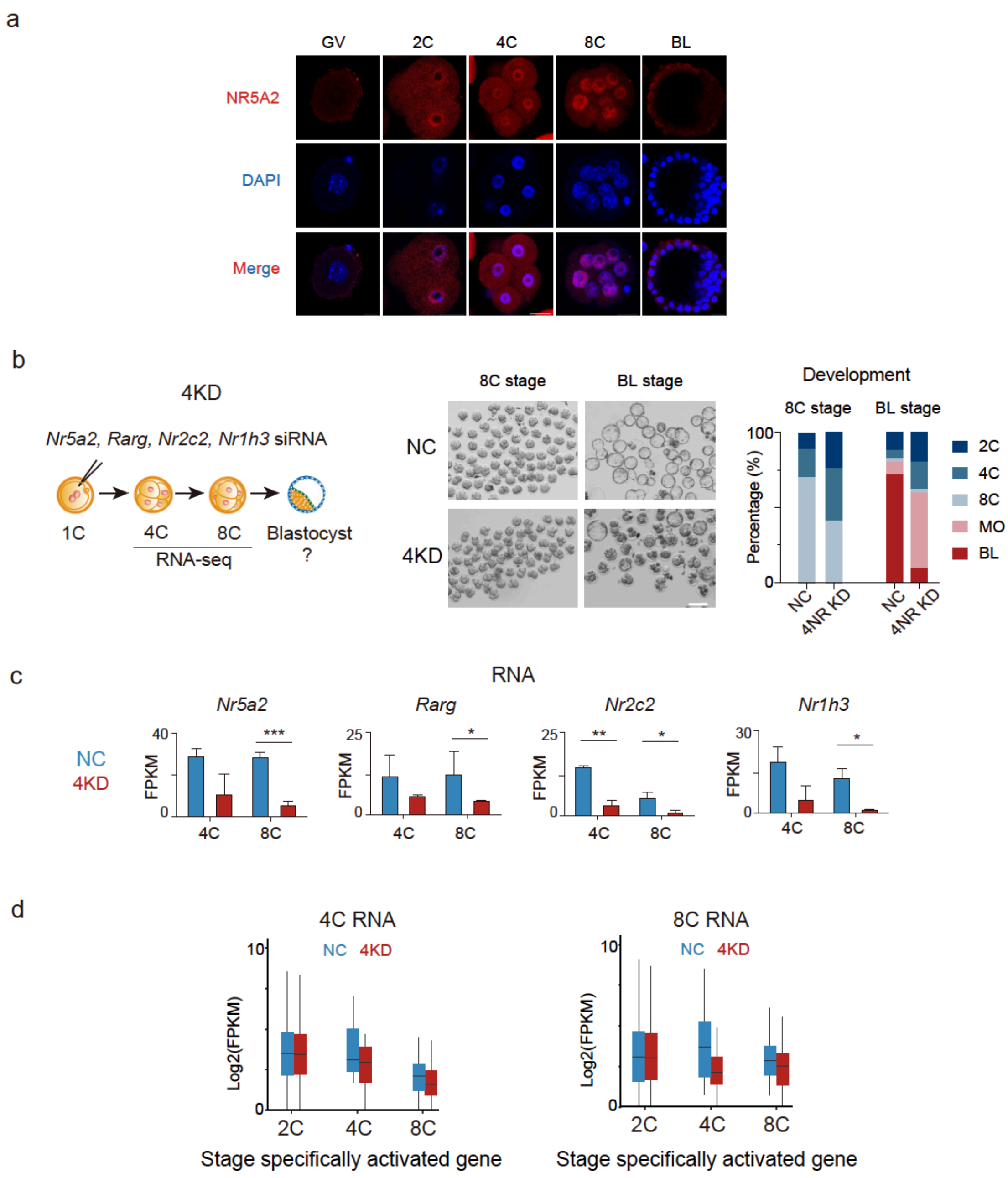

Supplementary information, Fig. S1. Joint knockdown of nuclear receptor TFs led to morula arrest. a, Immunofluorescence of NR5A2 (red) and DAPI (blue) in mouse germinal vesicle (GV) oocyte, L2C, 4C, 8C embryos, and blastocyst. Scale bar: 20 µm. **b,** Schematic of the combined knockdown of 4 ZGA-activated nuclear receptor factors (4KD, knockdown of *Nr5a2*, *Rarg*, *Nr1h3*, and *Nr2c2*) (left). Embryo morphology after knocking down 4 NR factors at the 8C and blastocyst stage (E4.5) (middle). Scale bar: 100 µm. Bar plots show the developmental rates of the NC group and 4KD group at the 8C and blastocyst stage (E4.5) (right). MO, morula; BL, blastocyst. **c,** Bar charts showing the RNA expression of 4 nuclear receptor factors in NC and 4KD group at the 4C and 8C stage based on RNA-seq. The error bars denote the standard deviations of two biological replicates of RNA-seq. **d,** Boxplots showing the average RNA expression levels of stage specifically activated genes in NC and 4KD groups at the 4C and 8C stages.
