# Supplementary material for: NR5A2 connects zygotic genome activation to the first lineage segregation in totipotent embryos
Source: Cell Res. 2023 Nov 7;33(12):952–66. doi: 10.1038/s41422-023-00887-z (PMC10709309; doi:10.1038/s41422-023-00887-z)
Supplement: Supplementary file 2 — Supplementary Fig. S2 [file 41422_2023_887_MOESM2_ESM.pdf]

Figure S2

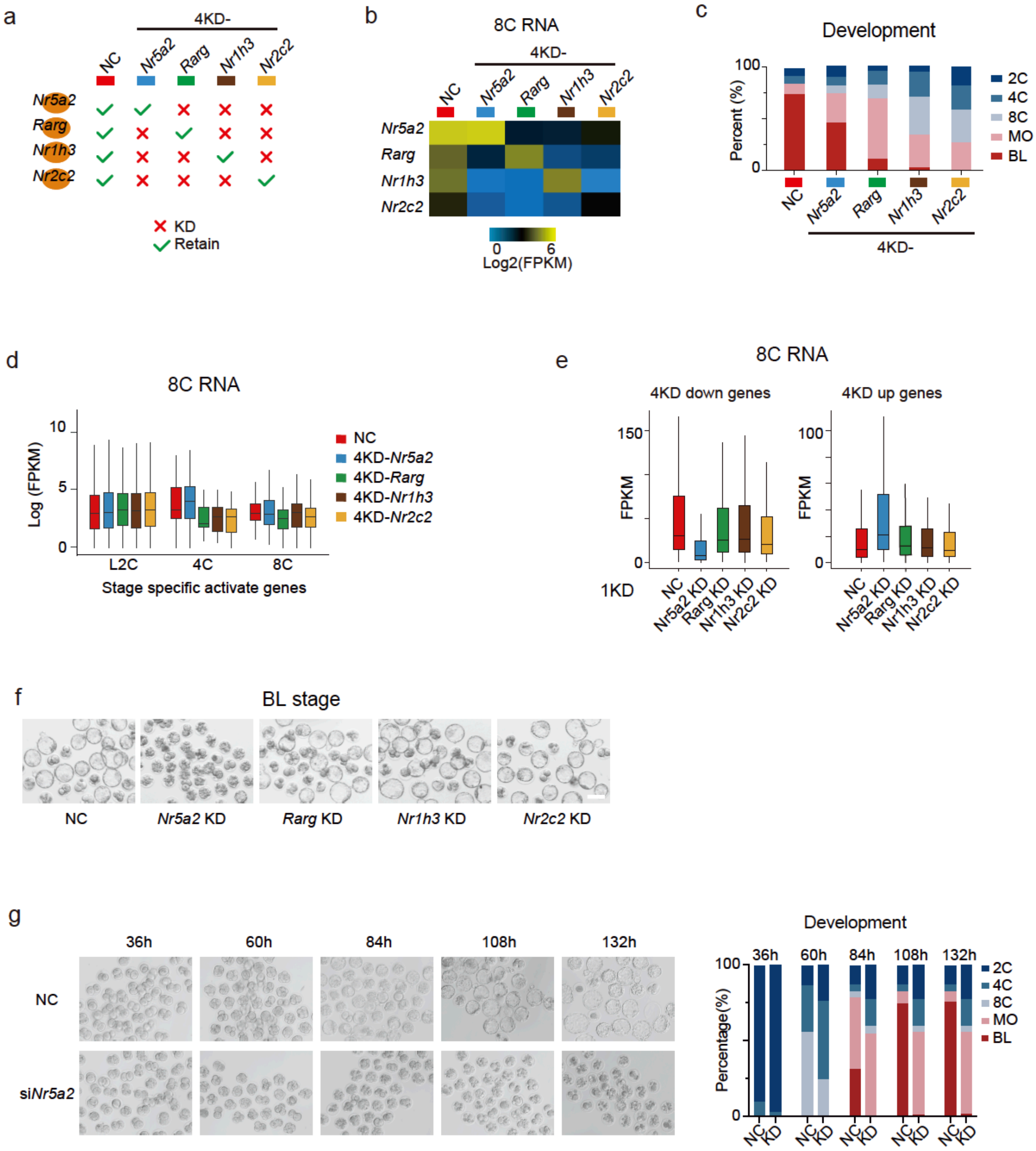

Supplementary information, Fig. S2. NR5A2 is the major functional factor among the 4 NR TFs activated during ZGA. a, Schematic illustration of the triple-knockdown (3KD, or 4 KD leave-one-out, 4KD-) among the 4 NR factors (*Nr5a2*, *Rarg*, *Nr1h3*, and *Nr2c2*). **b,** Heat maps showing RNA expression levels of each NR factor detected by RNA-seq in the NC group and each of the 3KD group at the 8C stage. **c**. Bar charts showing the developmental rates of NC and the 3KD groups at the blastocyst stage (E4.5). **d,** Box plots showing the average RNA expression levels of stage specifically activated genes of NC group (red) and each 3KD group at the 8C stage. **e,** Box plots showing the average RNA expression levels of down- (left) and up- (right) regulated genes of 4 KD in NC group (red), *Nr5a2* KD groups (blue), *Rarg* KD groups (green), *Nr1h3* KD groups (brown), and *Nr2c2* KD groups (yellow) at the 8C stage. **f,** Embryo morphology in NC group, *Nr5a2* KD groups, *Rarg* KD groups, *Nr1h3* KD groups, and *Nr2c2* KD groups at the blastocyst stage (E4.5). Scale bar: 100 µm. **g,** Embryo morphology of NC and *Nr5a2* KD group at 36h, 60h, 84h, 108h, and 132h after fertilization (left). Bar plots show the developmental rates of NC group and *Nr5a2* KD group at 36h, 60h, 84h, 108h, and 132h after fertilization (right).
