# Supplementary material for: NR5A2 connects zygotic genome activation to the first lineage segregation in totipotent embryos
Source: Cell Res. 2023 Nov 7;33(12):952–66. doi: 10.1038/s41422-023-00887-z (PMC10709309; doi:10.1038/s41422-023-00887-z)
Supplement: Supplementary file 3 — Supplementary Fig. S3 [file 41422_2023_887_MOESM3_ESM.pdf]

Figure S3

a

b

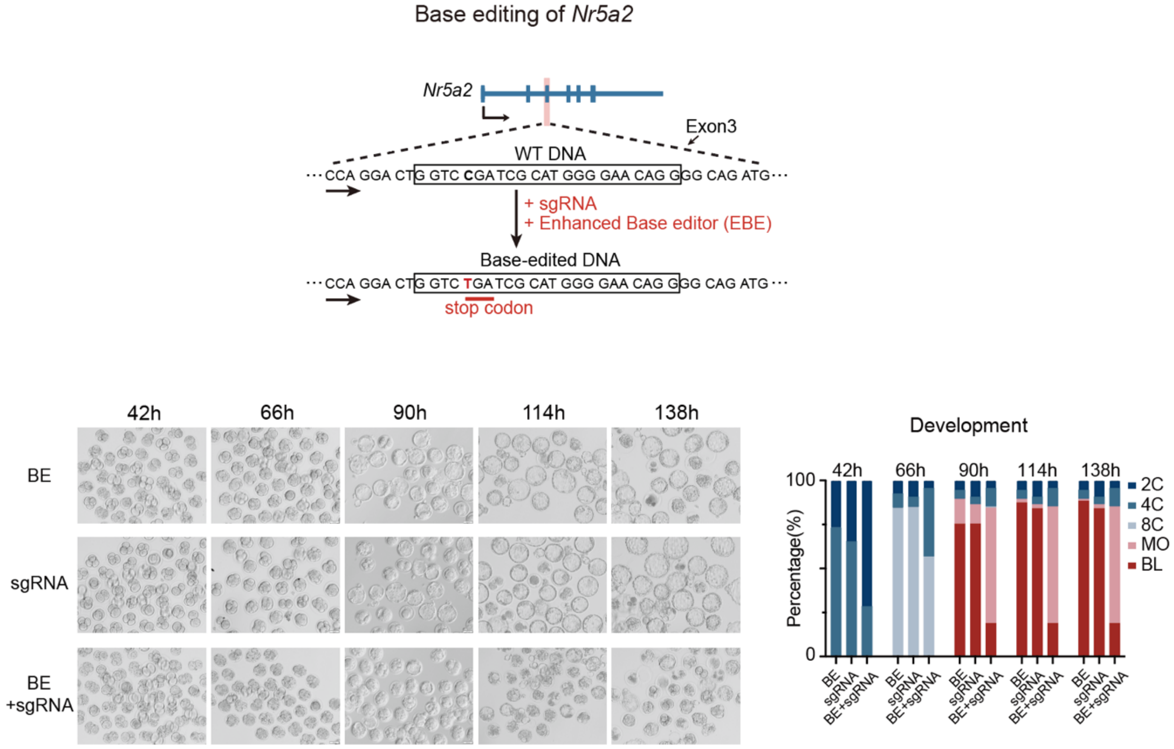

Supplementary information, Fig. S3. *Nr5a2* BE KO in mouse embryos. a, Schematic of base editing for *Nr5a2*. Exon 3 of *Nr5a2* is targeted. Box shows sgRNA targeting sequence (left, middle). An introduced stop codon is indicated. **b,** Embryo morphology after injection of *Nr5a2* sgRNA only, BE mRNA only, and both sgRNA and BE mRNA at 42h, 66h, 90h, 114h, and 138h after fertilization (left). Bar plots show the developmental rates after injection of *Nr5a2* sgRNA only, BE mRNA only, and both sgRNA and BE mRNA at 42h, 66h, 90h, 114h, and 138h after fertilization (right).
