# Supplementary material for: NR5A2 connects zygotic genome activation to the first lineage segregation in totipotent embryos
Source: Cell Res. 2023 Nov 7;33(12):952–66. doi: 10.1038/s41422-023-00887-z (PMC10709309; doi:10.1038/s41422-023-00887-z)
Supplement: Supplementary file 4 — Supplementary Fig. S4 [file 41422_2023_887_MOESM4_ESM.pdf]

## Figure S4

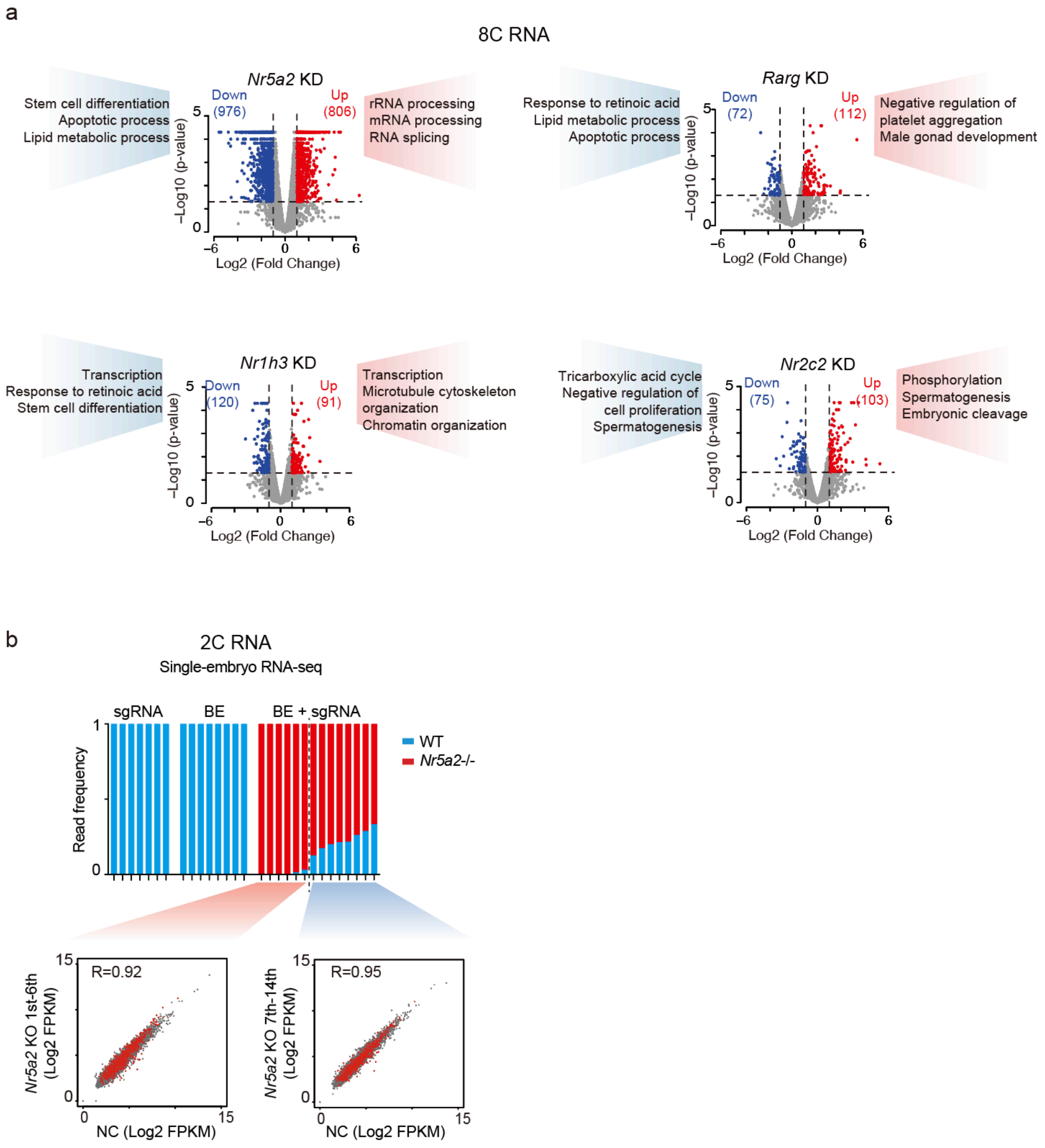


**Supplementary information, Fig. S4. Differentially expressed genes after the knockdown of NR factors. a,** Volcano plots showing differentially expressed genes in *Nr5a2* (top, left), *Rarg* (top, right), *Nr1h3* (bottom, left), or *Nr2c2* (bottom, right) single KD embryos at the 8C stage. Up-regulated (log2(Fold change)>2, P value<0.05) and down-regulated (log2(Fold change)>2, P value<0.05) genes are colored in red and blue, respectively. GO analysis results of differentially expressed genes are also shown. **b,** Bar charts showing the percentages of WT *Nr5a2* (blue) and base edited *Nr5a2* (red) reads from RNA-seq after injection of *Nr5a2* sgRNA only, BE mRNA only, and both sgRNA and BE mRNA at the 2C stage (top). Scatter plots compare gene expression of the NC group and *Nr5a2* KO groups with nearly-complete mutation rates (bottom left, 1$^{st}$-6$^{th}$ embryos) and high-to-medium mutation rates (bottom right, 7$^{th}$-14$^{th}$ embryos) in mouse 2C embryos. ZGA genes are colored in red. The Spearman correlation coefficients are also shown.
