# Supplementary material for: NR5A2 connects zygotic genome activation to the first lineage segregation in totipotent embryos
Source: Cell Res. 2023 Nov 7;33(12):952–66. doi: 10.1038/s41422-023-00887-z (PMC10709309; doi:10.1038/s41422-023-00887-z)
Supplement: Supplementary file 5 — Supplementary Fig. S5 [file 41422_2023_887_MOESM5_ESM.pdf]

Figure S5

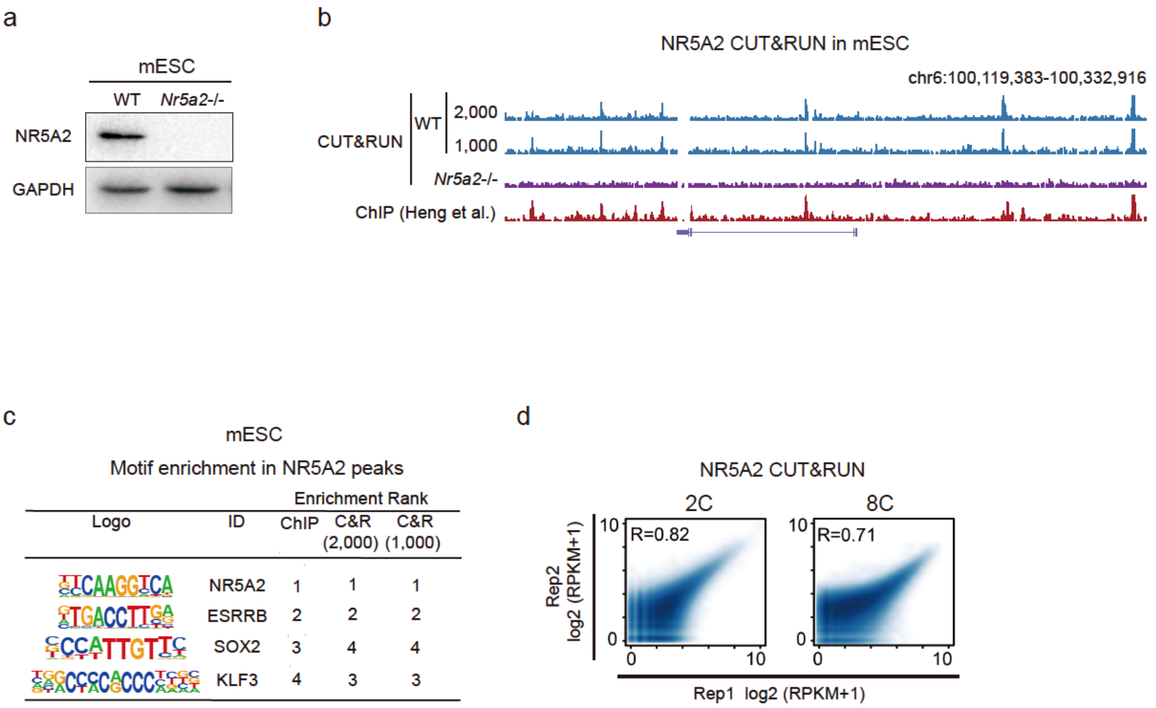

Supplementary information, Fig. S5. Validation of the NR5A2 CUT&RUN data. A, Western blot showing NR5A2 and GAPDH expression in WT and *Nr5a2*$^{-/-}$ mESCs. **B,** The UCSC browser view showing NR5A2 CUT&RUN and ChIP-seq [24] signals in WT mESCs (1,000 to 2,000 cells) and *Nr5a2*$^{-/-}$ mESCs (9,000 cells). **C,** Sequence logos and enrichment ranks of the motifs enriched in NR5A2 CUT&RUN peaks in mESCs. **D,** Scatter plots comparing the replicates of NR5A2 CUT&RUN data in mouse 2C and 8C embryos. The Pearson correlation coefficients are also shown.
