# Supplementary material for: NR5A2 connects zygotic genome activation to the first lineage segregation in totipotent embryos
Source: Cell Res. 2023 Nov 7;33(12):952–66. doi: 10.1038/s41422-023-00887-z (PMC10709309; doi:10.1038/s41422-023-00887-z)
Supplement: Supplementary file 6 — Supplementary Fig. S6 [file 41422_2023_887_MOESM6_ESM.pdf]

Figure S6

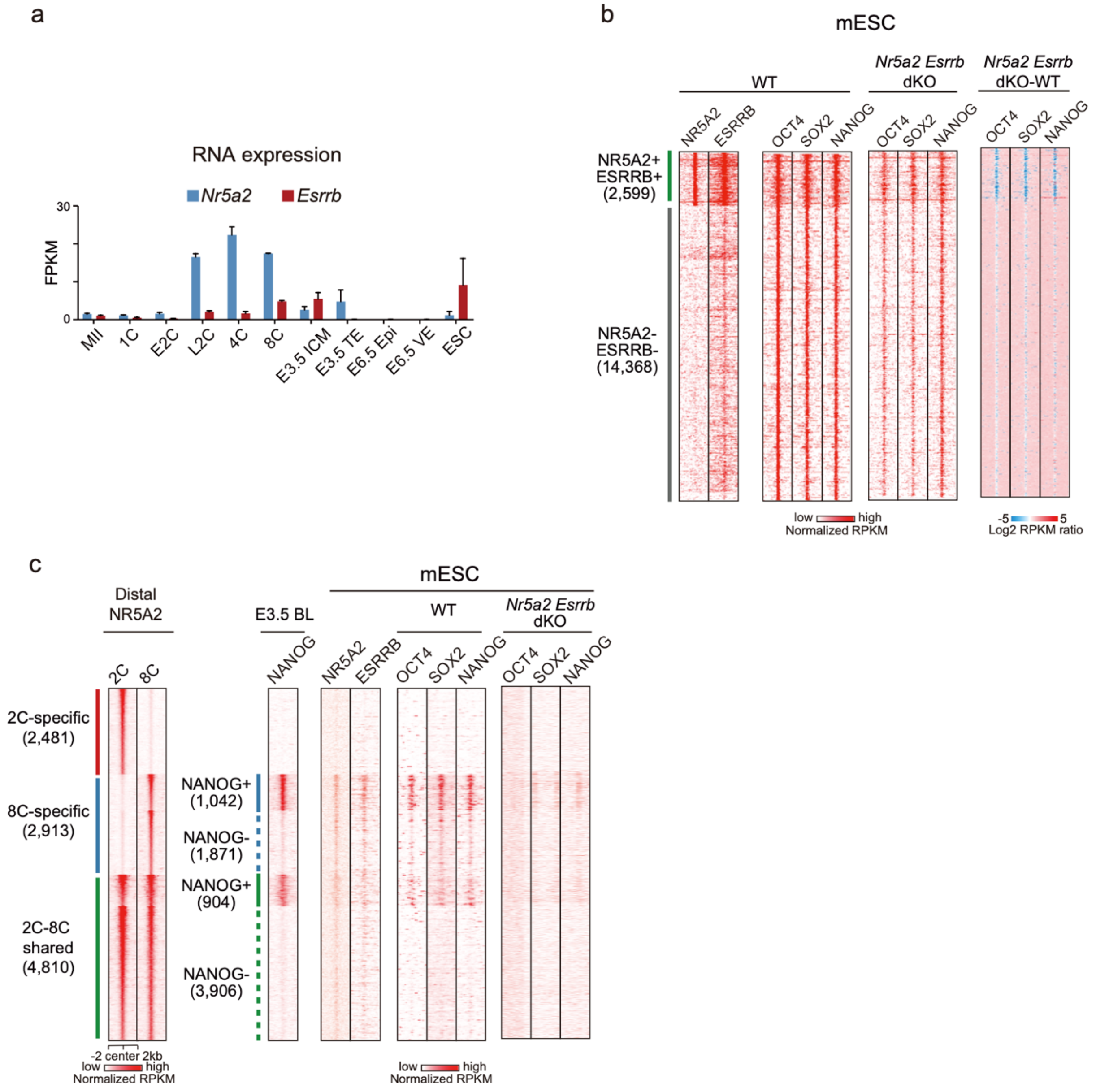

Supplementary information, Fig. S6. NR5A2 and ESRRB regulate the binding of NSO in mESCs. a, RNA expression (FPKM) of *Nr5a2* and *Esrrb*. The error bars denote the standard deviations of two biological replicates of RNA-seq. **b**, NSO binding sites (bound by all three factors) that are bound by both NR5A2 and ESRRB or bound by neither were selected. NR5A2 and ESRRB binding in WT mESCs, NSO binding in WT and *Nr5a2/Esrrb* double knockout (dKO) mESCs, and the differential binding of NSO between dKO and WT embryos are mapped. Data from Festuccia et al were re-analyzed and shown[27]. **c**, Heat maps showing NR5A2 binding at the 2C and 8C stages and NANOG binding in E3.5 blastocyst at the 2C-specific, 8C-specific, and 2C-8C shared NR5A2 distal binding peaks. Bindings of NR5A2 and ESRRB in WT mESCs, and NSO in WT and *Nr5a2/Esrrb* double knockout (dKO) mESCs are also mapped[27].
