# Supplementary material for: NR5A2 connects zygotic genome activation to the first lineage segregation in totipotent embryos
Source: Cell Res. 2023 Nov 7;33(12):952–66. doi: 10.1038/s41422-023-00887-z (PMC10709309; doi:10.1038/s41422-023-00887-z)
Supplement: Supplementary file 7 — Supplementary Fig. S7 [file 41422_2023_887_MOESM7_ESM.pdf]

Figure S7

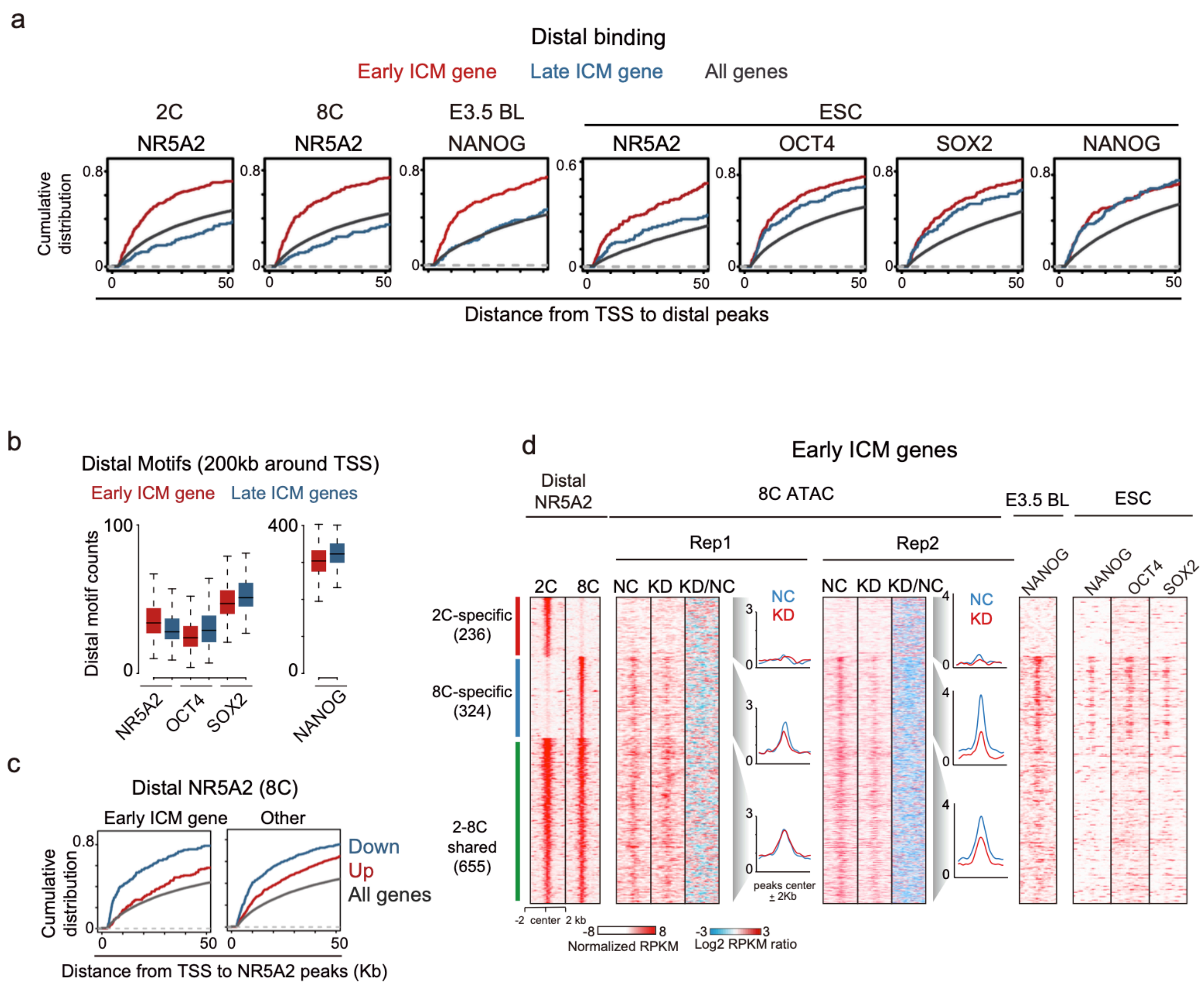

Supplementary information, Fig. S7. NR5A2 preferentially occupies and regulates early ICM genes. a, The cumulative distributions of early, late ICM, and all expressed genes with defined distances (x-axis) between their TSSs and nearest distal 2C, 8C, and ESC NR5A2 binding peaks, and OCT4, SOX2, and NANOG binding peaks in ESCs. **b,** Box plots showing the motif numbers of NR5A2, OCT4, SOX2, and NANOG in the distal regions (200kb around TSS, excluding TSS+/- 2.5kb) of early or late ICM genes. **c,** The cumulative distributions of down-regulated or up-regulated early ICM-specific, and other 8C-expressing genes in *Nr5a2* KD 8C embryos with defined distances (x-axis) between their TSSs and nearest distal 8-cell NR5A2 binding peaks. **d,** Heatmap showing the NR5A2 binding, enrichment of ATAC-seq signals in WT reference, NC, *Nr5a2* KD 8C embryos, and the ratios between *Nr5a2* KD and NC 8C embryos (left), average plots show enrichment of ATAC-seq signals in NC, *Nr5a2* KD 8C embryos (middle) at the 2C-specific, 8C-specific, and 2C-8C shared NR5A2 distal binding peaks near early ICM genes (250 Kb up- and down- stream of the TSSs). NANOG binding in E3.5 blastocyst and NANOG, OCT4, and SOX2 binding in ESC are also mapped and shown (right).
