# Supplementary material for: NR5A2 connects zygotic genome activation to the first lineage segregation in totipotent embryos
Source: Cell Res. 2023 Nov 7;33(12):952–66. doi: 10.1038/s41422-023-00887-z (PMC10709309; doi:10.1038/s41422-023-00887-z)
Supplement: Supplementary file 8 — Supplementary Fig. S8 [file 41422_2023_887_MOESM8_ESM.pdf]

Figure S8

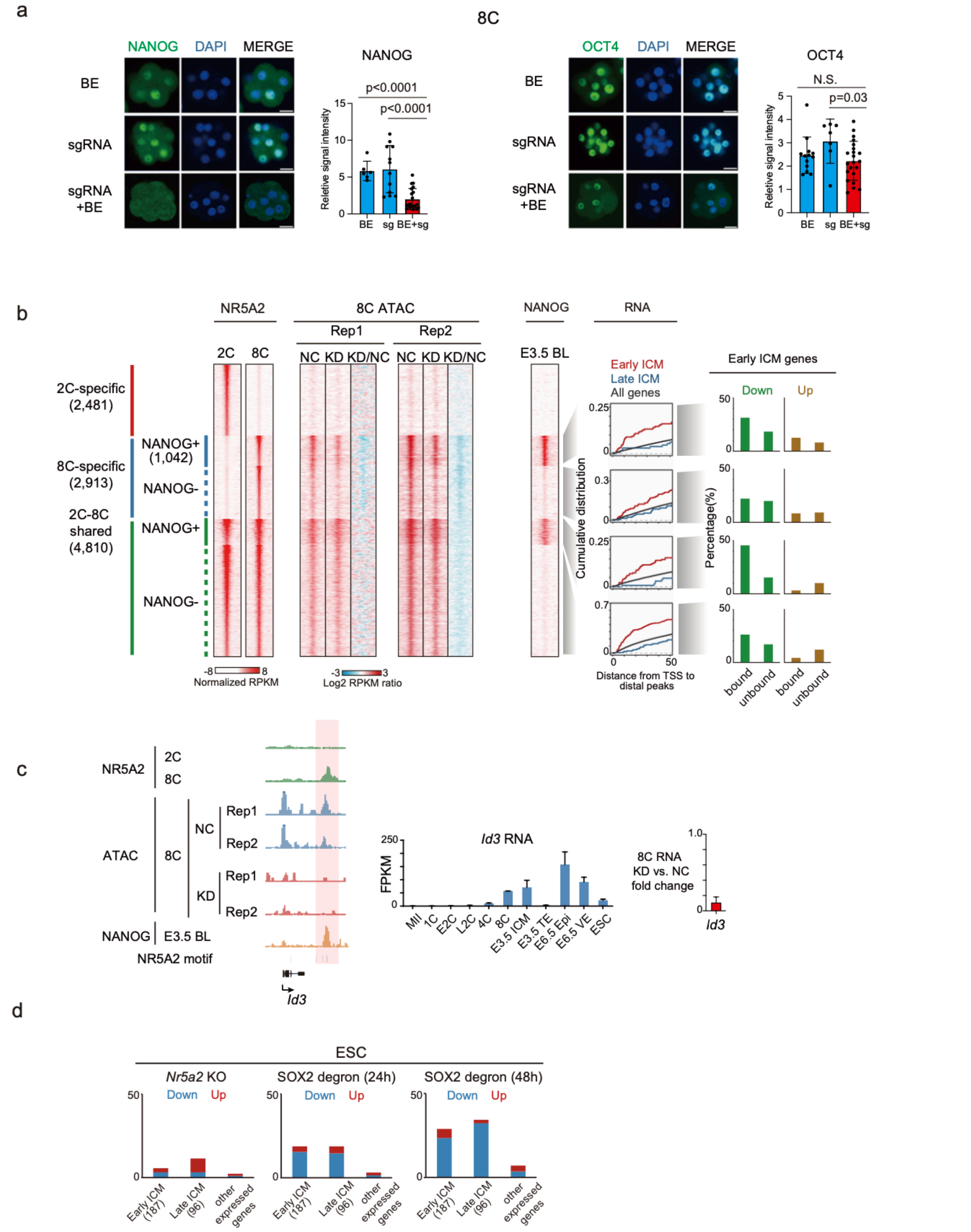

Supplementary information, Fig. S8. NR5A2 regulates early ICM genes. a, Immunofluorescence of NANOG (left) or OCT4 (right) (green), and DAPI (blue) in mouse 8C embryos after injection of *Nr5a2* sgRNA only, BE mRNA only, and both sgRNA and BE mRNA. Scale bar: 20 µm. Quantification of NANOG (n = 4-6 embryos) and OCT4 (n = 4-7 embryos)

signal intensity (relative to DAPI) and *P* values (t-test, two-sided) are shown. Each dot represents a single blastomere. **b,** Heat maps showing NR5A2 binding in 2C and 8C embryos and NANOG binding in E3.5 blastocyst (left). Cumulative plots show the distances of early ICM, late ICM, and all genes to the nearest distal 8-cell NR5A2 binding peaks (middle). Bar charts show the percentages of bound and unbound promoters of down- and up-regulated genes. **c,** The UCSC genome browser view showing NR5A2, ATAC, and NANOG signals near the *Id3* gene (left). Bar plot shows RNA expression (FPKM) across different stages (middle) and expression fold change (*Nr5a2* KD vs. control) in 8C embryos (right) for *Id3*. The error bars denote the standard deviations of two biological replicates of RNA-seq. **d,** Bar plots showing the percentages of down- and up-regulated genes among early, late ICM or other expressed genes in *Nr5a2* KO mESC (left), and SOX2-degron mESC after inducing SOX2 degradation for 24h (middle) or 48h (right).
