# Supplementary material for: NR5A2 connects zygotic genome activation to the first lineage segregation in totipotent embryos
Source: Cell Res. 2023 Nov 7;33(12):952–66. doi: 10.1038/s41422-023-00887-z (PMC10709309; doi:10.1038/s41422-023-00887-z)
Supplement: Supplementary file 9 — Supplementary Fig. S9 [file 41422_2023_887_MOESM9_ESM.pdf]

Figure S9

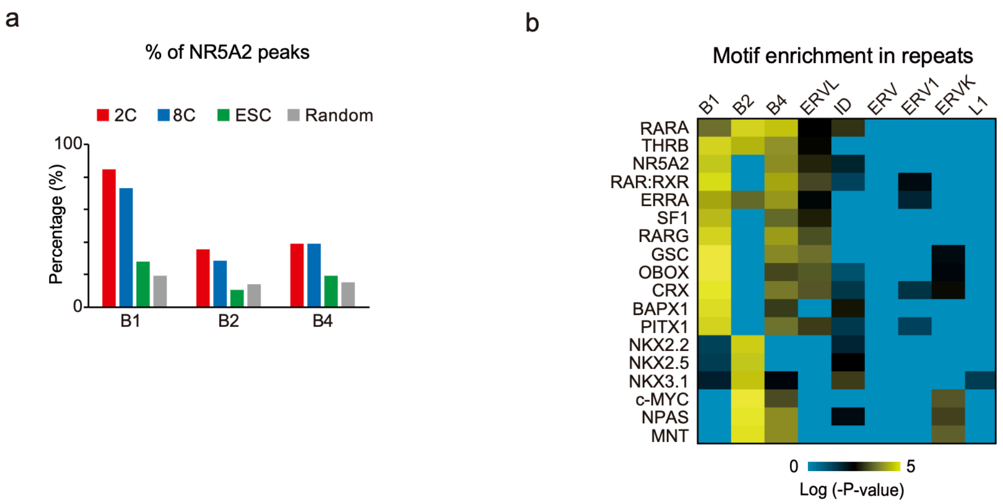

Supplementary information, Fig. S9. NR5A2 binding sites in early embryos enrich for transposable elements. a, Bar charts showing the percentages of NR5A2 peaks overlapped with B1, B2, and B4 repeats in 2C (red), 8C (blue), and ESC (green). Random peaks were shuffled and generated with lengths matched, and were similarly analyzed. **b,** Heat maps showing TF motif enrichment in different groups of repeats.
