# Supplementary material for: NR5A2 connects zygotic genome activation to the first lineage segregation in totipotent embryos
Source: Cell Res. 2023 Nov 7;33(12):952–66. doi: 10.1038/s41422-023-00887-z (PMC10709309; doi:10.1038/s41422-023-00887-z)
Supplement: Supplementary file 10 — Supplementary Fig. S10 [file 41422_2023_887_MOESM10_ESM.pdf]

Figure S10

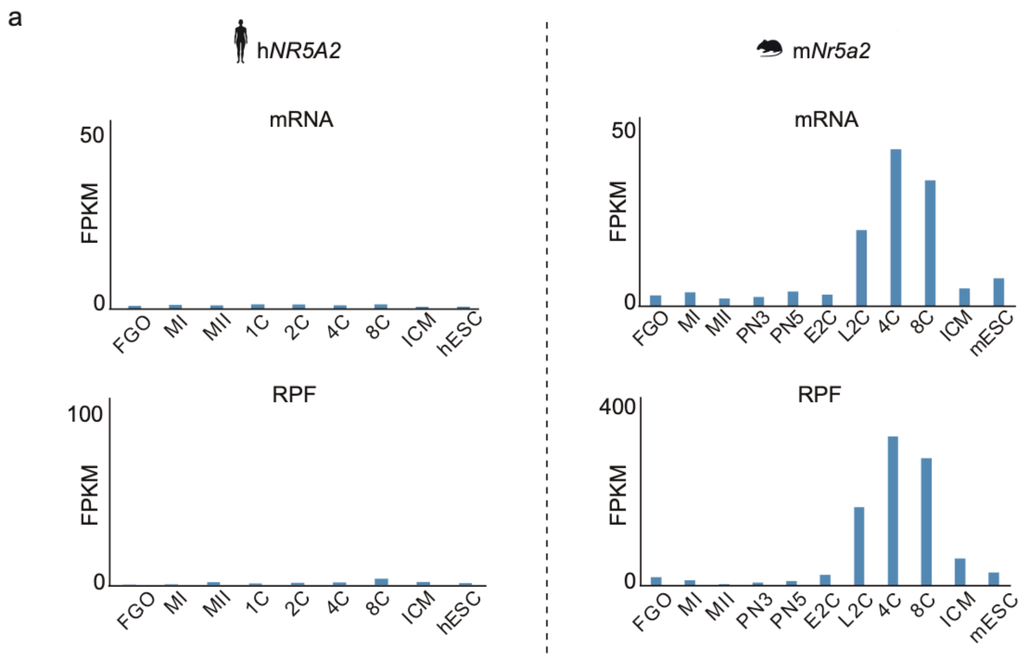

Supplementary information, Fig. S10. *NR5A2* expression in human and mouse early embryos. a, Bar charts showing *NR5A2/Nr5a2* mRNA levels from RNA-seq and ribosome-protected fragment (RPF, reflecting translation) levels from Ribo-seq in human (left) and mouse (right) early embryos.[58, 59]
